# The Efficacy Comparison Between Guan-Fu Base A Hydrochloric Injection vs. Propafenone Hydrochloric Injection in the Treatment of Arrhythmia: Systemic Review and Meta-Analysis

**DOI:** 10.3389/fcvm.2021.723932

**Published:** 2021-11-05

**Authors:** Jinming Song, Yao Tang, Chao Gao, Xiaofeng Hou, Xinyue Liu, Zongpei Xu

**Affiliations:** ^1^Tianjin University of Traditional Chinese Medicine, Tianjin, China; ^2^First Teaching Hospital, Tianjin University of Traditional Chinese Medicine, Tianjin, China

**Keywords:** Guan-Fu base A, propafenone, arrhythmia, ventricular premature beats (VPB), supraventricular tachycardia (SVT)

## Abstract

**Objective:** To determine using a systematic assessment and meta-analysis if GFA injection is an appropriate substitute of propafenone for arrhythmic.

**Design:** Conduct a systematic review and meta-analysis of randomized controlled trials.

**Data Source:** PubMed, Web of Science, Cochrane Library, Embase, Wan-Fang Database, VIP, CNKI, and Sino Med from their inception to 7 March 2021.

**Eligibility Criteria for Selecting Studies:** Inclusion of randomized controlled trials, which draws a comparison between GFA and propafenone. Evaluation of study integrity and conducted an extraction of independent data.

**Main Outcome Measure:** Efficacy for supraventricular tachycardia, it is considered effective if it is reversed within 40 min (without considering recurrence); for premature ventricular beats, if they are reduced by more than 50% within 6 h.

**Results:** Included in this current study are 1,294 research subjects pooled from 14 clinical studies. From the pooled assessment, GFA is demonstrated to be the equivalent of propafenone regarding the potency of effectiveness for tachycardia (RR = 1.11, 95% CI: 0.96, 1.28, *P* = 0.15). The subset analysis indicated that GFA has a better effect on premature ventricular beats (RR = 1.35, 95% CI: 1.07, 1.70, *P* = 0.01) and a similar effect on supraventricular tachycardia (RR = 1.07, 95% CI: 0.98, 1.12, *P* = 0.21). GFA effectiveness is lesser than propafenone in the case of mean converting time (WMD = −1.18, 95% CI: −2.30, −0.07, *P* = 0.04), systolic blood pressure (WMD = −3.53, 95% CI: −6.97, −0.09, *P* = 0.04), and QRS complex (WMD = −3.82, 95% CI: −6.96, −0.69, *P* = 0.02). Both GFA and propafenone have identical effects for diastolic blood pressure, heart rate, P-R interval, and QTc interval.

**Conclusion:** A meta-analysis of RCTs was performed across 14 clinical trials, whereby 1,294 patients are used as research subjects. From the results, it is revealed that the effect exhibited by GFA injection is similar to the propafenone injection when treating premature ventricular beats or supraventricular tachycardia. Nevertheless, in certain academic disciplines, it was found that GFA is safer and beneficial compared to propafenone. Based on facts from relevant studies, GFA is deemed applicable during clinical practice.

**Systematic Review Registration:** https://www.inplasy.com/inplasy-2021-3-0077/, identifier: INPLASY202130077.

## Introduction

Arrhythmia is the consequence of fluctuation in the electrophysical features of the heart, which is inclusive of excitability, autonomy, conductivity, refractory period, and repolarization (1). In general, antiarrhythmic drugs are categorized into 5 divisions on the basis of classification proposed by Vaughan Williams. According to the mode of mechanism, the drug migrates across the cell membrane and is chemically bound to receptors of the resting, activated, and inactivated channels within the membrane. During the reaction of those drugs, it subsequently triggered a spectrum of negative effects (such as cardiac and extracardiac toxicity). Thus, it becomes expedient to find arrhythmic drugs that could serve as safe alternatives (2). As regards the GFA, it is a novel form of alkaloid having an antiarrhythmic effect. It is extracted from aconitum root, which is a conventional medicine of Chinese origin, and its effect is identical to that of class 1 antiarrhythmic drugs (3, 4). GFA has been confirmed from preclinical studies that it exhibits an inhibitory effect on various ion channels, for instance, sodium channel current in ventricular myocytes and L-type calcium channel current, and delayed after potassium current. What is more is that it also can slow down the spontaneous frequency of the sinus node and reduces the rapid response action potential amplitude and maximum depolarization speed, prolonging the action potential duration and effective refractory period. Under experimental conditions, the new drug has the potential of counteracting supraventricular, ventricular tachycardia, ventricular fibrillation, atrial flutter, and atrial fibrillation (5). The China Food and Drug Administration (CFDA) in 2005 granted the approval of GFA to treat arrhythmia. Therefore, we aimed to assess the current evidence regarding the effectiveness of GFA on SVT and VPB for human being.

Seven prominent forms of SVT exist, which are listed as follows: the sinus tachycardia, atrial fibrillation, atrial flutter, supraventricular tachycardias, atrioventricular nodal reentrant tachycardia, atrioventricular reciprocating (reentrant) tachycardia atrial tachycardia, and multifocal atrial tachycardia (6). From a statistical perspective, the supraventricular patients are approximately 2.25 per thousand individuals, and its incidence case on an annual basis is about 35 cases per 100,000 people (7). Concerning patients diagnosed with arrhythmias, their average expenditure per year is nearly doubled the amount before diagnosis, thereby conferring a considerable financial burden on the healthcare system (8). SVT is generally treated using the following methods namely: vagal maneuvers, administration of adenosine, intravenous injection of class Ic or class III antiarrhythmic drugs (e.g., propafenone, sotalol, or amiodarone), electrical cardioversion, or catheter ablation (9).

When there is an abnormal focus of discharge or loops in the ventricular myocardium, it resulted in ventricular tachycardia, which could occur in myocardial ischemia, fixed structural heart disease as a result of previous myocardial infarction or non-ischemic disease, ion channel disease or in the heart functioning normally, bringing about hemodynamic changes with the prospect of advancing into sudden cardiac death in the worst-case scenario (10). Treatment methods such as pharmacological treatment, catheter ablation, or interventional (e.g., left ventricular assist device and cardiac resynchronization therapy) are used for the general treatment (11–13).

Recently, regarding the GFA effect when treating tachyarrhythmia, the existing large-scale multi-center randomized controlled study of GFA is not sufficient. Being a novel drug, it still lacks a confirmed result as regards its direction on its application and feasibility in clinical application. Thus, it is crucial to set up a detailed assessment of current clinical evidence in finding answers to the question posed. Nevertheless, as far as GFA is concerned, it is yet to witness a systematic review or meta-analyses. In order to ensure a more appropriate clinical decision is made and determine potential research areas that still need to be explored, this current study is conducted for evaluating the potency of GFA for both SVT and VPB using a systematic review and meta-analysis of RCTs.

## Methods

### Design

The study design was conducted in accordance with the 2009 preferred reporting guidelines to conduct systematic reviews and meta-analysis (PRISMA) statements (14). Our review protocol was prospectively registered with INPLASY (INPLASY202130077).

### Study Selection Criteria

#### Search Strategy

From the initial stage of the study, the two authors (Song J. and Tang Y) conducted fact-finding for literature review from CNKI, Wan Fang, VIP, Sino med, PubMed, Web of Science, Embase, and Cochrane Library, which lasted until March 7, 2021, in the absence of language barriers. Furthermore, the researchers searched through ClinicalTrials.gov along with https://www.chictr.org.cn/. The basis of the search strategy is the PICOS principle, which combines subject words and free words. The identification of these contents is actualized following repeated inspections and supplemented by manual search along with tracking of citation of these papers at the appropriate time.

To ensure the removal of articles that fail to meet eligibility criteria during the initial procedure, two same reviewers screened the entire paper abstracts. The eligible articles are subsequently evaluated and obtained in full text by the same set of two evaluators, who conducted an independent assessment of each article, and data extraction and quality assessment. Resolution of disputes was resolved via third-party discussion.

#### Criteria of the Included Literature

1. For the trials, the participants used are patients suffering from VPB or SVT without critical structural disease of the heart.
2. As for the basic information, no statistically significant difference exists.
3. RCTs.
4. Included as outcome indicators are primary indicators or one of the secondary outcomes.

#### Criteria of the Excluded Literature

1. Arrhythmia that is caused through other underlying conditions includes anemia, hyperthyroidism, etc.
2. The two groups of studies are not corresponding with the illness baseline level regarding their health status.
3. The experiment is designed as an observational experiment or an interventional experiment in which a control group is not included.
4. The outcome indicators are vague.

#### Data Extraction

From the eligible studies, the following data are extracted namely: (1) First author's name, (2) Publication year, (3) Region, (4) Design, (5) Disease type (either SVT or VPB), (6) Intervention, (7) Dosage, (8) Duration of observation, (9) The number of participants, (10) age, (11) Gender, and (12) Outcomes.

#### Intervention Measures

As for SVT, the GFA of 140 mg/10 ml was used, and the duration for completing the injection was within 5 min at a dose of 4 mg/kg. As for the control group, a propafenone of 35 mg/10 ml was applied, and the duration of completion of the injection was within 5 min at a dose of 1 mg/kg. In case the effect of the first dose applied is not apparent, then after 15 min, a second dose will be injected. The method of injecting is identical to the first injection applied. Overall, the entire procedure lasted for 40 min. For the experimental group, GFA of 140 mg/10 ml was used in treating VPB. Following the administration of an initial dose of 4 mg kg^−1^, it was maintained intravenously at 20 μg kg^−1^ min^−1^. The propafenone is loaded at a dosage of 1 mg kg^−1^ and was maintained intravenously at 5 μg kg^−1^ min^−1^. After 6 h of sustained observation, the number of VPB was recorded.

#### Primary Outcomes

Efficacy is the primary outcome. For the SVT to be adjudged as effective, the heart rhythm must be converted to sinus rhythm within 40 min of administering medication (inclusive of successfully converted and subsequent relapsed), and if this conversion is not observed, then it is considered ineffective. Regarding VPB, it is considered effective if there is a reduction in the premature beats by not <50% following administration of medication. It would be considered ineffective assuming they fail to attain the effective level.

#### Secondary Outcomes

Included in the secondary outcomes were blood pressure such as systolic and diastolic blood pressure, ECG indicators, such as HR, QRS, PR, and QTc, and mean converting time. Supposing it is not feasible to extract secondary outcomes from a study, then during the stage of analysis where those outcomes were involved, that particular study will be removed from the analysis.

### Quality Assessment

In the selected study, Cochrane criteria were used in performing the systematic assessment of bias. For the assessment, the following items are used: random sequence generation, allocation concealment, blinding of participants and personal, blinding of outcome assessment, incomplete outcome data, selective reporting, and other biases (15). Two reviewers (Song J. and Tang Y) conducted an independent risk-of-bias assessment. Misunderstanding of opinions would be resolved through a consensual-oriented discussion.

### Data Synthesis

Stata (version 15.1) was used in conducting meta-analysis. As regards binary variables, RR and 95% CI were utilized for assessing the combined effect, continuous variables use WMD and 95% CI, and *P* ≤ 0.05 is considered statistically significant. By applying the Higgins index (*I*^2^) and the Chi-square test to assess heterogeneity. Assuming *I*^2^ < 40% and Chi-square test *P* > 0.1, then no heterogeneity exists; the results are combined via the fixed-effects model (utilizing the M-H method for binary variables, the variance method inverse for continuous variables). Assuming *I*^2^ ≥ 40% or Chi-square test *P* > 0.1 and the cause of the heterogeneity is unclear, a random-effect model (D + L method) is applied.

### Additional Analysis

In consideration of the different diseases (SVT and VPB), subgroup assessments were conducted. In addition to the subgroup assessment, qualitative analysis of HR, QRS, P-R, QRS, and QTc was equally carried out.

### Meta-Regression Analysis

For exploring the heterogeneity, fixed effects meta-regression analyses inclusive of certain potential independent moderator variables (e.g., age at baseline and gender) were planned. In both arms, with the exemption of studies having zero events, meta-regression analyses were performed. Two-sided *P* ≤ 0.05 was adjudged to have significance.

### Publication Bias

The visual inspection of Begg's funnel plot asymmetry was used in exploring the potential publication biases, while the potential publishing bias is explored using the quantitative test that uses Egger's weighted regression test and Begg's adjusted rank correlation test. Moreover, the analysis on the publication bias is adjusted using Duval and Tweedie's “trim and fill” method. Two-sided *P* ≤ 0.05 was deemed to be significant.

## Results

### Eligible Studies and Study Characteristics

**Figure 1:**
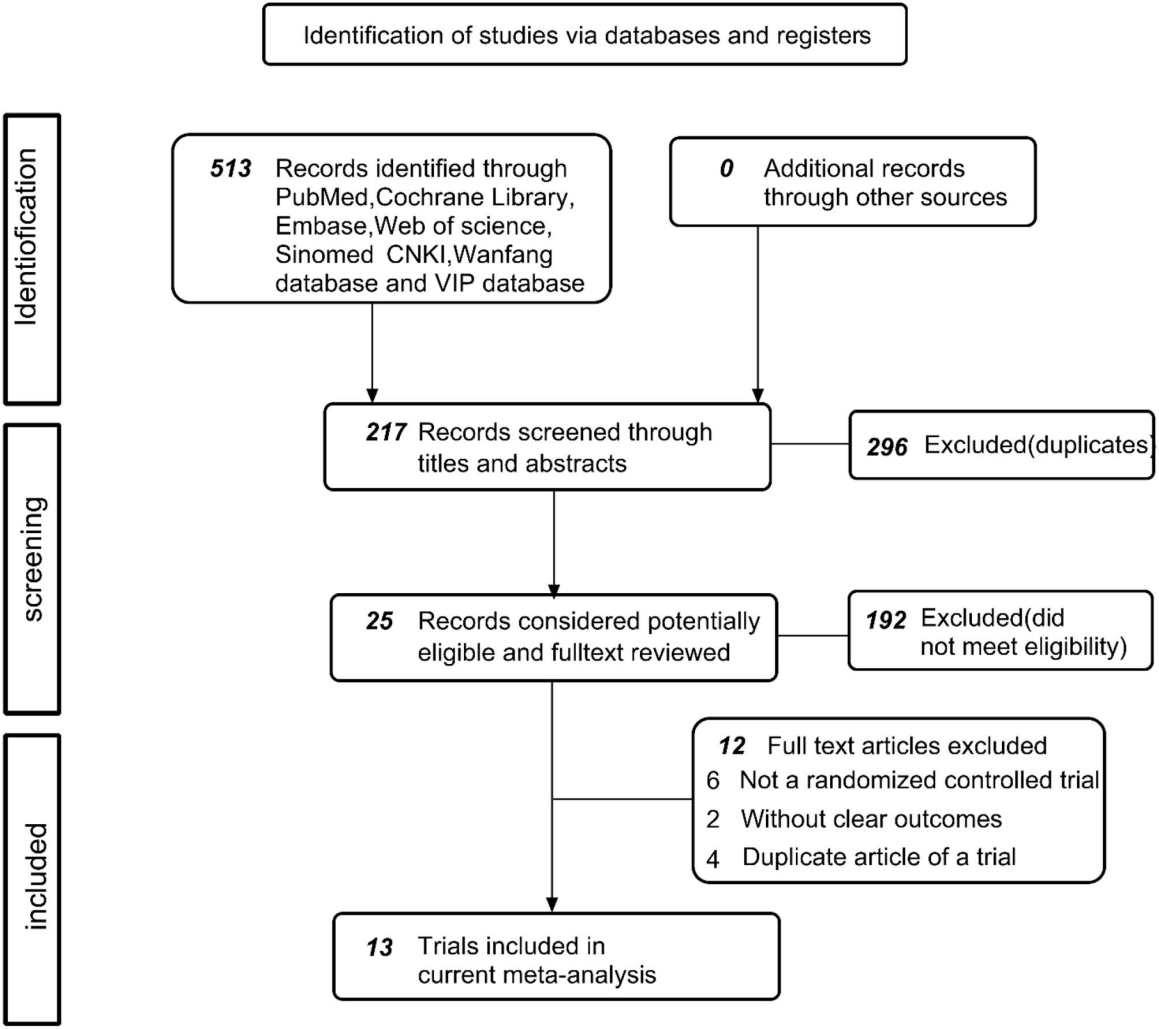
Flow chart of the number of studies identified and included into the meta-analysis.

In the first instance, the identification of 513 documents was conducted, including 296 duplicate documents and 192 reviews, animal experiments, meta-analyses, pharmacological experiments, etc. Eventually, a total of 25 original texts was cautiously assessed and subjected to review. Among the articles were no control group (*n* = 7) (15–21), publication that is repeated (*n* = 4) (22–25), and the unclear or ambiguous experimental content (*n* = 1) (26). Finally, 13 articles (included 14 trials) (24, 27–39) were justified as being eligible and included a meta-analysis. The procedure for research is illustrated in Figure 1.

The source of data collection is from 14 randomized controlled clinical trials and 28 treatment groups where the study participants are 1,306 individuals (included 2 people withdrew before the trial), whereby 776 are in the experimental group (GFA) and 528 belong to the control group (propafenone). The trial is completed by a total of 1,294 individuals, 12 individuals dropped out of the trial, and those who dropped out were excluded from the final assessment.

The literature included in this study is the one published from 2003 to 2019 and all the publications are from China. Approximately, 10 studies were on supraventricular tachycardia and 4 studies of ventricular tachycardia. The summary of features of the studies reviewed is presented in Table 1.

**Table 1:** Characteristics of included studies.

**Study**	**Year**	**Region**	**Design**	**Type**	**Intervention**	**Dosage regimen**	**Observation duration**	**N**	**Age (year)**	**Gender**	**Outcome**
Zhihong Han (1)	2003	China	Randomized parallel-group study	SVT	GFA vs. Propafenone	GFA hydrochloride (140 mg/10 ml) 4 mg/kg completed in 5 min; Propafenone injection (35 mg/10 ml) 1 mg/kg completed in 5 min.	40 min	32/8	48.4 ± 12.9	13/27	(1)(4)(6)(8)(9)(12)
Xin Gao (3)	2004	China	Randomized double-blind parallel-group study	SVT	GFA vs. Propafenone	GFA hydrochloride (140 mg/10 ml) 4 mg/kg completed in 5 min; Propafenone injection (35 mg/10 ml) 1 mg/kg completed in 5 min.	40 min	30/32	49.3 ± 12.4	33/29	(1)(3)(4)(5)(6)(7)(8) (9)(10)(11)(12)(14)
Qiji Geng	2006	China	Randomized double-blind parallel-group study	SVT	GFA vs. Propafenone	GFA hydrochloride (140 mg/10 ml) 4 mg/kg completed in 5 min; Propafenone injection (35 mg/10 ml) 1 mg/kg completed in 5 min.	40 min	23/22	50.6 ± 14.1	27/18	(1)(3)(4)(5)(6)(7) (8)(10)(11)(12)
Xin Gao (1)	2007	China	Multicenter Randomized double-blind parallel-group study	SVT	GFA vs. Propafenone	GFA hydrochloride (140 mg/10 ml) 4 mg/kg completed in 5 min; Propafenone injection (35 mg/10 ml) 1 mg/kg completed in 5 min	40 min	101/100	47.4 ± 14.0	100/101	(1)(6)(7)(8)(9) (10)(11)(12)
Xin Gao (2)	2007	China	Multicenter, Randomized parallel-group study	SVT	GFA vs. Propafenone	GFA hydrochloride (140 mg/10 ml) 4 mg/kg completed in 5 min; Propafenone injection (35 mg/10 ml) 1 mg/kg completed in 5 min.	40 min	137/ 46	43.2 ± 13.1	60/77	(1)(10)(12)(13)(14)
Aihui Wu	2010	China	Randomized parallel group study	SVT	GFA vs. Propafenone	GFA hydrochloride (140 mg/10 ml) 4 mg/kg completed in 5 min; Propafenone injection (35 mg/10 ml) 1 mg/kg completed in 5 min.	40 min	50/50	50:45:00	64/36	(1)(13)
Jin Lin	2010	China	Randomizeddouble-blind, parallel-group study	SVT	GFA vs. Propafenone	GFA hydrochloride (140 mg/10 ml) 4 mg/kg completed in 5 min; Propafenone injection (35 mg/10 ml) 1 mg/kg completed in 5 min.	40 min	41/41	42.10 ± 16.62	51/31	(1)(3)(4)(10)(12)
Xian yong Zheng	2012	China	Parallel-group study	SVT	GFA vs. Propafenone	GFA hydrochloride (140 mg/10 ml) 4 mg/kg completed in 5 min; Propafenone injection (35 mg/10 ml) 1 mg/kg completed in 5 min.	40 min	54/67	45.76 ± 14.53	55/66	(1)(3)(4)(5)(6) (7)(8)(10)(12)
Yanwei Wang	2014	China	Randomized, parallel-group study	SVT	GFA vs. Propafenone	GFA hydrochloride (140 mg/10 ml) 4 mg/kg completed in 5 min; Propafenone injection (35 mg/10 ml) 1 mg/kg completed in 5 min.	40 min	31/32	—	—	(1)(3)(4)(5)(6) (8)(9)(10)(12)
Ao Wei	2019	China	Randomized, parallel-group study	SVT	GFA vs. Propafenone	GFA hydrochloride (140 mg/10 ml) 4 mg/kg completed in 5 min; Propafenone injection (35 mg/10 ml) 1 mg/kg completed in 5 min.	40 min	43/43	47.67 ± 3.98	45/41	(1)(10)
Lianfang Chang	2004	China	Randomized, parallel-group study	SVT	GFA vs. Propafenone	GFA (140 mg/10 ml)4 mg·kg^−1^;maintained intravenously at 20 μg·kg^−1^·min^−1^. Propafenone (35 mg/10 ml) 1 mg·kg^−1^;maintained intravenously at 5 μg·kg^−1^·min^−1^	6 h	42/14	37.6 ± 12.5	17/39	(2)(10)
Xin Gao (4)	2004	China	Randomized, double-blind, parallel-group study	SVT	GFA vs. Propafenone	GFA(140 mg/10 ml)4 mg·kg^−1^;maintained intravenously at 20 μg·kg^−1^·min^−1^. propafenone(35 mg/10 ml) 1 mg·kg^−1^; maintained intravenously at 5 μg·kg^−1^·min^−1^	6 h	20/21	44.7 ± 11.0	11/30	(2)(10)
Zhihong Han (2)	2003	China	Randomized, parallel-group study	VPB	GFA vs. Propafenone	GFA (140 mg/10 ml)4 mg·kg^−1^;maintained intravenously at 20 μg·kg^−1^·min^−1^. Propafenone (35 mg/10 ml) 1 mg·kg^−1^; maintained intravenously at 5 μg·kg^−1^·min^−1^	6 h	28/7	52.8 ± 12.5	13/22	(2)(3)(4)(6)(8)(9)(10)
Yanmin Yang	2006	China	Multicenter, Randomized, parallel-group study	VPB	GFA vs. Propafenone	GFA (140 mg/10 ml)4 mg·kg^−1^;maintained intravenously at 20 μg·kg^−1^·min^−1^. Propafenone (35 mg/10 ml) 1 mg·kg^−1^; maintained intravenously at 5 μg·kg^−1^·min^−1^	6 h	144/47	45.7 ± 14.0	64/127	(2)(10)

*Outcomes: (1) Efficacy (The number of patients with SVT who had reversed in 40 min); (2) Efficacy (Number of VPB 6 h after drug administration) (3) heart rate; (4) Blood pressure; (5) P-wave; (6) P-R interval; (7) R-R interval; (8) QRS wave complex; (9) QTc interval; (10) Adverse effect; (11) Sinus node recovery time; (12) Mean time of converting; (13) Recurrence rate in 24 h; (14)The number of 15 min recurrence; (15) Average dosage*.

### Risk of Bias Assessment

Nearly all studies selected were characterized through adequate information on sequence generation, allocation concealment, and staff and outcome evaluations, and through incomplete outcome data and selective reporting of outcomes indicated a low risk of bias. Table 2 presented the quality of the bias assessment in detail.

**Table 2:** Risk of bias assessment.

**Study**	**Year**	**Random** **sequence** **generation**	**Allocation** **concealment**	**Blinding of** **participants** **and personal**	**Blinding of** **outcome** **assessment**	**Incomplete** **outcome** **data**	**Selective** **reporting**	**Other** **bias**
Zhihong Han (1)	2003	U	U	H	H	L	U	U
Xin Gao (3)	2004	L	L	L	L	L	U	L
Qiji Geng	2006	L	L	L	H	L	U	L
Xin Gao (1)	2007	L	U	U	H	L	U	U
Xin Gao (2)	2007	L	U	H	H	L	U	L
Aihui Wu	2010	L	L	U	H	L	U	U
Jin Lin	2010	L	L	L	L	L	U	U
Xianyong Zheng	2012	L	U	L	H	L	U	L
Yanwei Wang	2014	U	U	U	H	L	U	U
AoWei	2019	L	U	U	H	L	U	L
Zhihong wHan (2)	2003	L	U	L	H	L	U	L
Lianfang Chang	2004	U	L	U	H	L	U	U
Xin Gao (4)	2004	L	L	U	H	L	U	U
Yanmin Yang	2006	L	L	H	H	L	U	U

*H, high risk of bias; U, unclear risk of bias; L, low risk of bias*.

### Meta-Analysis Results

#### Primary Outcome: Efficacy

**Figure 2:**
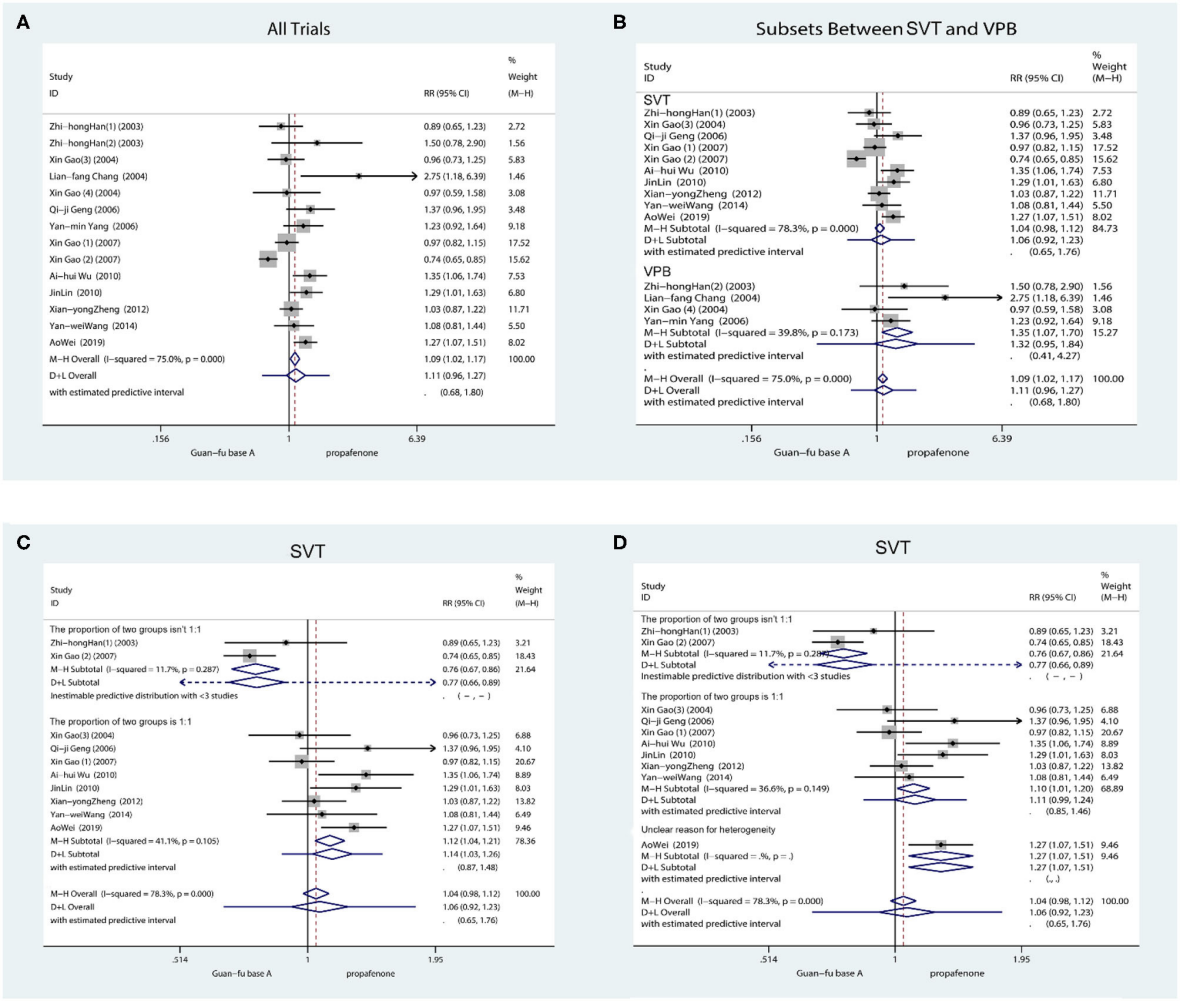
Forest plot comparing the GFA with propafenone in efficiency. **(A)** Forest plot comparing the GFA with Propafenone for all study. **(B)** Forest plot comparing the GFA with Propafenone in subgroups (SVT and VPB). **(C)** Forest plot comparing the GFA with Propafenone for SVT without articles of “Zhihong Han (1)” and “Gao Xin (2).” **(D)** Forest plot comparing the GFA with Propafenone for SVT without articles of Xin Gao (1).

The 14 trials indicated the number of individuals who had converted to a normal rhythm of SVT or reduction of VPB. From the outcome, it is shown that the efficacy between the GFA group and the propafenone group is not significant from a statistical perspective (RR = 1.11, 95% CI: 0.96, 1.27, *P* = 0.15). According to the heterogeneity test (*I*^2^ = 75.0%, *P* < 0.001), the random method was accepted. Subsequently, from the subgroup assessment of SVT and VPB, the GFA was found to be more appropriate for treating VPB compared to propafenone (RR = 1.35, 95% CI: 1.07, 1.70, *P* = 0.01), the heterogeneity test (*I*^2^ = 39.8%, *P* = 0.17). Nevertheless, strong heterogeneity (*I*^2^ = 78.3%, *P* < 0.001) is still shown by the SVT group (RR = 1.07, 95% CI: 0.98, 1.12, *P* = 0.21) (Figure 2). Subsequent research revealed that the participants' numbers in two articles in the experimental group and the control group are significantly different. As regards the number of subjects, the number of individuals in the test group reached three times than of those in the control group in “Zhi-hong Han (1)” and “Gao Xin (2).” It is revealed from further meta-regression analysis that the number between the experimental group and the control group actually caused the heterogeneity (*P* = 0.007, adjusted *R*^2^ = 78.62%) (Supplementary Figure 1). If both articles are not included, the test for heterogeneity is *I*^2^ = 41.1%, *P* = 0.11. From the sensitivity analysis with the leave-one-out method (Supplementary Figure 4), it is demonstrated that “Xin Gao (1)” differs from other studies; however, the cause of this is vague. Therefore, the D + L method was applied (RR = 1.14, 95% CI: 1.03, 1.26, *P* = 0.015). From the results, GFA is shown to have a more appropriate effect when treating SVT. Subsequent meta-regression analysis of RR on age and sex orientation/gender ratio (men: women) found that the treatment of the SVT group was not age-dependent (*P* = 0.48 > 0.05) (Supplementary Figure 2) and related to sex orientation (Coef = 0.44, SD = 0.13, 95% CI: 0.12, 0.72, *P* = 0.01) (Supplementary Figure 3), demonstrating that GFA appears to have a more effective impact on SVT in men. The funnel plot showed potential publication bias (Supplementary Figure 5); the Egger's test result (SVT: Coef = 2.48, SD = 1.14, 95% CI: −0.04, 4.98; *P* = 0.05; VPB: Coef = −0.12, SD = 0.36, 95% CI: −1.66, 1.42, *P* = 0.40) (Supplementary Figures 6, 8) and Begg's test (SVT: Scores = 3, SD = 11.18, *P* = 0.86; VPB: Score = 4, SD = 2.94, *P* = 0.31) (Supplementary Figures 7, 9) showed unpublished publication bias. In the case of SVT and VPB, correcting the asymmetry through the application of the “Trim and Fill” method revealed four potentially missing pieces of literature on the left side of the funnel plot, reducing the estimated effect to 0.91 (95% CI: 0.78, 1.07). As regards VPB, method 1 revealed a possibly missing study on the left side of the funnel plot; the estimated effect is reduced to 1.13 (95% CI: 0.81, 1.58) (Supplementary Figures 10, 11).

#### Secondary Outcome: Blood Pressure

**Figure 3:**
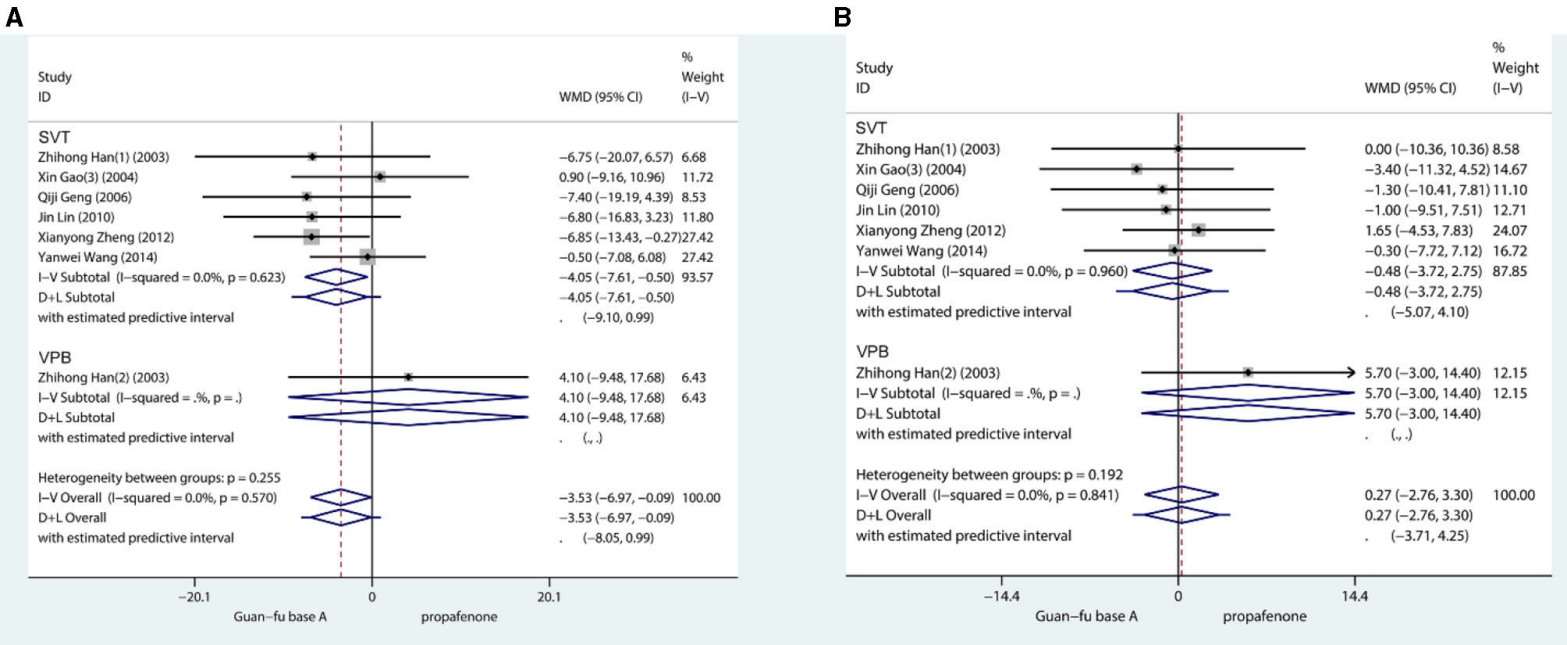
Forest plot comparing the GFA with propafenone in blood pressure. **(A)** Forest plot comparing the GFA with Propafenone in systolic blood pressure. **(B)** Forest plot comparing the GFA with Propafenone in diastolic blood pressure.

Approximately 7 trials in total recorded blood pressure before and after the medication had been administered. The result shows that when compared with the propafenone group, the SBP was lower in the experimental group (WMD = −3.53, 95% CI: −6.97, −0.09, *P* = 0.04) (Figure 3). The combined effect of DBP showed that the differences between the two groups lacked statistical significance (WMD = 0.27, 95% CI: −2.76, 3.30, *P* = 0.84). If SBP is compared with the GFA group before and after the experiments, the result appears to have marginally increased. Comparing the DBP before and after in two groups, they were approximately equivalent. No glaring publication bias is shown in the funnel chart; from the Begg's test and Egger's test, it is not indicated that any potential publication bias exists [SBP: (Begg: Score = 9, SD = 6.66, *P* = 0.23; Egger: Coef = 0.36, SD = 1.33, 95% CI: −3.06, 3.79, *P* = 0.796)] [DBP: (Begg:Score = −1, SD = 6.66, *P* = 1.00; Egger: Coef = −0.23, SD = 1.82, 95% CI: −4.91, 4.45, *P* = 0.905)] (Supplementary Figures 12–17).

#### Secondary Outcome: ECG Indicators

**Figure 4:**
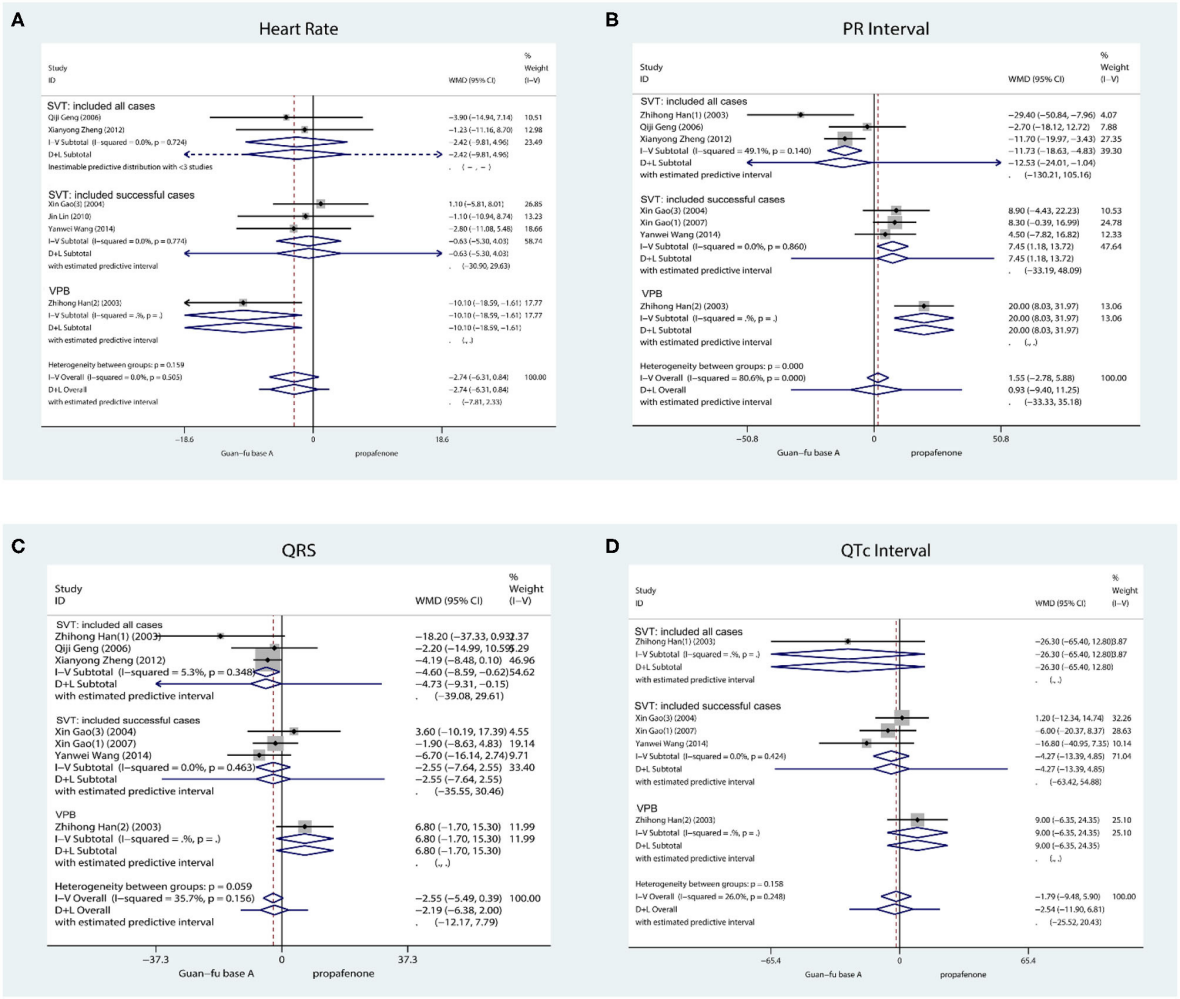
Forest plot comparing the GFA with Propafenone in ECG indicator. **(A)** Forest plot comparing the GFA with Propafenone in HR. **(B)** Forest plot comparing the GFA with Propafenone in PR. **(C)** Forest plot comparing the GFA with Propafenone in QRS complex. **(D)** Forest plot comparing the GFA with Propafenone in QTc interval.

HR: Six studies took the record of the HR prior to and following the administration of medication. The first one was ventricular tachycardia while the second was supraventricular tachycardia. From the result, the treatment effect was not statistically different between the two groups (WMD = −2.74, 95% CI: −6.14, 0.8, *P* = 0.13) (Figure 4). The heterogeneity test *I*^2^ = 0.0%, *P* = 0.59, funnel plots, Begg's test (Score = −5, SD = 6.66, *P* = 0.55), and Egger's test indicate that no publication bias exists (Coef. = −0.88, SD = 1.40, 95% CI: −4.49, 2.73, *P* = 0.56) (Supplementary Figures 18–20).

P-R: The record of the PR interval is taken by about 7 studies in total, 3 studies investigated successful cases, and 3 studies investigated all cases. It is revealed from their combined effect that the effect of GFA is similar to propafenone in P-R (WMD = 0.93, 95% CI: −9.40, 11.25, *P* = 0.48) (Figure 4) due to *I*^2^ = 80.6%, *P* < 0.001, method D + L. The funnel plot and the Begg's (Score = −5, SD = 6.66, *P* = 0.55) and Egger's tests (Coef = −2.19, SD = 2.76, 95% CI: −9.89, 5.49, *P* = 0.26) indicated that no apparent publication bias exists (Supplementary Figures 21–23).

QRS: Approximately 7 studies recorded QRS prior to and once medication had been administered. One study was for ventricular arrhythmia and three for supraventricular arrhythmia. The combined effect indicates that the GFA group was slightly smaller than the propafenone group during the prolonged QRS duration in SVT, and the difference is significant from a statistical perspective (WMD = −3.82, 95% CI: −6.96, −0.69, *P* = 0.02, *I*^2^ = 0.0%, *P* = 0.54; Figure 4). No apparent publication change exists by observing the funnel plots, Begg's test, and Egger's test (Coef = −0.53, SD = 0.53, 95% CI: −2.84, 1.78, *P* = 0.43) (Supplementary Figures 24–26).

QTc: QTc is recorded by a total of 5 studies and they were all supraventricular arrhythmias. The result whose assumption is that no statistical significance exists between the GFA group and propafenone group integrated with inverse variance method (WMD = −5.41, 95% Cl: −14.29, 3.45, *P* = 0.23, *I*^2^ = 0.0%, *P* = 0.41) (Figure 4). When considering the visual observation of funnel plot and the consequence of Begg's (Score = −4, SD = 4.08, *P* = 0.46) and Egger's test (Coef = −2.37, SD = 1.29, 95% Cl: −6.48, 1.73, *P* = 0.163), it is apparent that publication bias does not exist (Supplementary Figures 27–29).

#### Secondary Outcome: Mean Time of Converting

**Figure 5:**
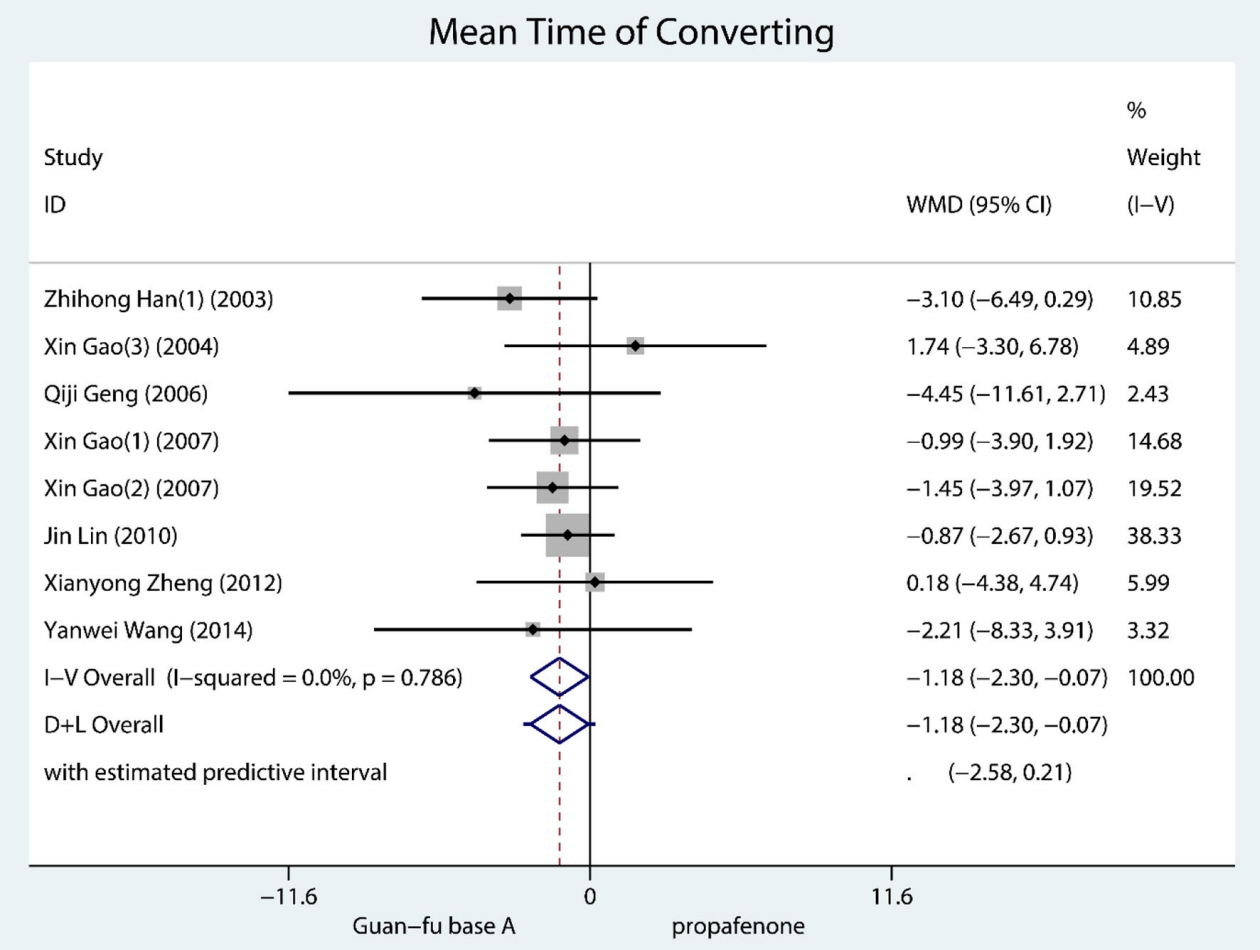
Forest plot comparing the GFA with propafenone in mean converting time.

A total of 8 trials documented mean time of converting and they were all SVT. According to the result, GFA seems to have worked better than propafenone for resuscitation (WMD = −1.18, 95% CI: −2.30, −0.07, *P* = 0.04, *I*^2^ = 0.0, *P* = 0.79) (Figure 5). No considerable publication bias is shown in the funnel chart; from the Begg's test and Egger's test, it is not indicated that any potential publication bias exists (Supplementary Figures 30–32).

## Discussion

Through the action on inactivated and resting states of *I*_*Na*_,GFA inhibits *I*_*Na*_ in a concentration as well as frequency-dependent manner (40). Frequency dependence (the drug will have a stronger inhibitive effect if the channel opening has more frequency) could serve as the main mechanism of the drug when treating arrhythmias, and the drug's selective inhibitory activity on late sodium currents could have effectiveness in the prevention or treatment of arrhythmias and improving the pumping function of the cardiac. Additionally, GFA can reduce the spontaneous rate of the sinus node to the normal range and conduction in the atrium and junction areas, thereby increasing the time frame of action potentials (41). In addition, GFA at high doses affects ventricular repolarization and prolongs ventricular depolarization, thus inhibiting *I*_Ca,L_, and *I*_k_ at reduced dosages. The weak effect of GFA on *I*_Ca,L_ is applicable for patients with heart failure and bradycardia because of its slight negative inotropic effect (4). Besides the impact on cardiac ion channels, it has been revealed from certain research that the antiarrhythmic effects of guanfacine may likewise be related to inhibition of oxidative stress damage (42) and non-endothelial-dependent diastolic effects (43). Further experiments have revealed that the elimination half-life of GFA is 480.4 ± 134.4 min; however, the elimination half-life of injected propafenone is approximately 3.5 to 4 h (44). GFA inhibits human CYP2D6 as approximately 20% active drug in clinical practice and is metabolized in the liver. When GFA is applied in clinical practice, clinicians need to take drug interactions into consideration, including selective serotonin reuptake inhibitors, tricyclic antidepressants, as well as antipsychotics drugs, etc. (45).

The electrophysiological action of propafenone is in the inhibition of the *I*_Na_ of the heart muscle and Purkinje fiber, non-actively inhibits *I*_k_ and β-Adrenergic receptors, reduces cardiac arousal, and inhibits spontaneous self-regulation and activated activity. EKG performance reduces HR, increases PR and QRS, and slightly increases the time duration of QT. The most important hemodynamic function is the negative inotropic effect on the heart, which leads to the possible worsening of symptoms of left ventricular failure as well as congestive cardiac insufficiency (2, 46).

From this meta-analysis, it is revealed that GFA and propafenone showed no statistical difference in its level of efficacy in treating tachyarrhythmia and that GFA at this dose was comparable to propafenone in terms of derivative lowering and increased ventricular repolarization. Thus, the effect of GFA on the treatment of tachyarrhythmias requires further study. In comparing GFA and propafenone in mean converting time and electrophysiological indicators with HR, PR, QRS, and QTc, the results assume that GFA takes effect more quickly and has a smaller influence on QRS. In view of the results, GFA may reduce the number of side effects, including induced sudden cardiac death caused by complicated expansion of the QRS (47, 48). When comparing the antihypertensive effects of two groups, GFA had a relatively weaker effect on blood pressure. The result can be explained by the mechanism of two drugs. Propafenone is crucial in the inhibition of the β-adrenergic receptor to lower blood pressure, but GFA acts on the vascular endothelium as a non-blocking β-adrenergic receptor. Furthermore, it took an abnormal appearance to mention that after the end of the injection, systolic blood pressure was high in two groups (Supplementary Figure 33). Diastolic value was not high, which was inconsistent with the pharmacological effects of propafenone and GFA. The literature fails to mention the reason for the increase and could be as a result of drug interactions or incorrect measurements.

## Limitations of the Study

(1) There is a wide variation in the quality of the studies included in these trials and some of the literature was not sufficiently precise to establish inclusion and exclusion criteria. They failed to differentiate between atrial supraventricular tachycardia or atrioventricular fold tachycardia or atrioventricular fold tachycardia and did not report if patients had bypass and atrioventricular block that could add to the difference in efficacy between studies.
(2) Baseline values between studies were vague, nothing is reported concerning the duration of illness and seizures in patients in any of the studies, and whether appropriate medication use was stopped for more than 5 half-lives, which could have resulted in significant differences between studies.
(3) In the included studies, only immediate indicators were observed and no detailed observations were made on the relapse after administration of the drug and its effects on the pathological process of the patient, so the long-term effect of the drug is unknown.
(4) In the previous experiments, different doses of the drug worked differently and no articles on the topic have conducted a subsequent investigation on this matter.

Conclusively, a meta-analysis of RCTs is performed across 14 trials, which involves 1,306 patients, and from the results, it is indicated that GFA is comparable in efficacy to propafenone when treating ventricular thrombosis or supraventricular tachycardia. Contrastingly, GFA is less negative than propafenone in terms of negative inotropic effects. GFA has been confirmed from the result to be considered in clinical practice during cardioversion with tachyarrhythmia drugs.

## Data Availability Statement

The original contributions presented in the study are included in the article/Supplementary Material, further inquiries can be directed to the corresponding author.

## Author Contributions

All authors listed have made a substantial, direct and intellectual contribution to the work, and approved it for publication.

## Conflict of Interest

The authors declare that the research was conducted in the absence of any commercial or financial relationships that could be construed as a potential conflict of interest.

## Publisher's Note

All claims expressed in this article are solely those of the authors and do not necessarily represent those of their affiliated organizations, or those of the publisher, the editors and the reviewers. Any product that may be evaluated in this article, or claim that may be made by its manufacturer, is not guaranteed or endorsed by the publisher.

## Abbreviations

SVT: supraventricular tachycardia
VPB: ventricular premature beats
GFA: Guan-Fu base A
RR: relative risk
BP: blood pressure
HR: heart rate
PR: P-R interval
QRS: QRS complex
QT: QT interval
QTc: corrected QT interval
INa.: sodium channel current
INa.L: late sodium current
INa.T: transient sodium current
IHERG: HERG current
IKV1.5: KV1.5 current
ICa,L: L-type calcium current
Ik: the delayed rectifier potassium current
H: high risk of bias
U: unclear risk of bias
L: low risk of bias
CI: confidence interval
SD: standard difference
WMD: weighted mean difference
SMD, standard mean difference: GFA: Guan-Fu base ahydrochloride injection
propafenone: propafenone hydrochloride injection
RCTs: randomized controlled trials.
