# Supplementary material for: The Efficacy Comparison Between Guan-Fu Base A Hydrochloric Injection vs. Propafenone Hydrochloric Injection in the Treatment of Arrhythmia: Systemic Review and Meta-Analysis
Source: Front Cardiovasc Med. 2021 Nov 5;8:723932. doi: 10.3389/fcvm.2021.723932 (PMC8602695; doi:10.3389/fcvm.2021.723932)
Supplement: Supplementary file 1 [file Data_Sheet_1.PDF]

Supplementary table 1.

**Supplementary Table 1. full Search terms and strategy used for systematically reviewing the articles**		
**No**	**Concept**	**Search terms**
#1	GFA injection	(Acehytisine[Title/Abstract]) OR (Kwan-fu base A[Title/Abstract]) OR (Guan-fu base A[Title/Abstract]) OR (Guanfu base A[Title/Abstract]) OR (Guanfubase A[Title/Abstract]) OR (Kwan-fu base A[Title/Abstract]) OR (Acehytisine Hydrochloride[Title/Abstract]) OR (Acehytisine Hydrochloride injection[Title/Abstract]) OR (GFA[Title/Abstract])
#2	Arrhythmia	"arrhythmia cardiac"[Title/Abstract] OR "cardiac dysrhythmia"[Title/Abstract] OR "dysrhythmia cardiac"[Title/Abstract] OR "cardiac arrhythmia"[Title/Abstract] OR "cardiac arrhythmias"[Title/Abstract] OR "Arrhythmia"[Title/Abstract] OR "Arrythmia"[Title/Abstract] OR "supraventricular tachycardia"[Title/Abstract] OR "supraventricular tachycardias"[Title/Abstract] OR "tachycardias supraventricular"[Title/Abstract] OR "ventricular tachycardias"[Title/Abstract] OR "ventricular tachycardia"[Title/Abstract] OR "ventricular tachyarrhythmias"[Title/Abstract] OR "tachyarrhythmia ventricular"[Title/Abstract] OR "ventricular tachyarrhythmia"[Title/Abstract] OR "paroxysmal supraventricular tachycardia"[Title/Abstract] OR "paroxysmal supraventricular tachycardias"[Title/Abstract] OR "supraventricular tachycardia paroxysmal"[Title/Abstract] OR "tachycardia paroxysmal supraventricular"[Title/Abstract] OR "nonsustained ventricular tachycardia"[Title/Abstract] OR "nonsustained ventricular tachycardias"[Title/Abstract] OR "tachycardia nonsustained ventricular"[Title/Abstract]
3	Combination	#1 and #2

Supplementary table 2.

**Supplementary Table 2. full Search terms and strategy used for systematically reviewing the articles. (in Chinese database)**		
**No**	**Concept**	**Search terms**
#1	GFA injection	"阵发性房室结折返性室上性心动过速"[常用字段]) AND "关附甲素"[常用字段] OR "关白附子甲素"[常用字段] OR "盐酸关附甲素"[常用字段] OR "达芬齐芯"[常用字段]
#2	Arrhythmia	"阵发性室上性心动过速"[常用字段] OR "心律失常"[常用字段] OR "快速型心律失常"[常用字段] OR "室性心律失常"[常用字段] OR "心动过速"[常用字段] OR "心动过速"[常用字段] OR "阵发性房室折返性心动过速"[常用字段] OR "阵发性房室结折返性室上性心动过速"[常用字段] OR "室性心动过速" OR "室性早搏"[常用字段]
3	Combination	#1 and #2

Supplementary Figure 1. Meta-regression bubble plots of the association between log RR and Proportion of participants between GFA and Propafenone. The size of each circle is inversely proportional to the variance of change.

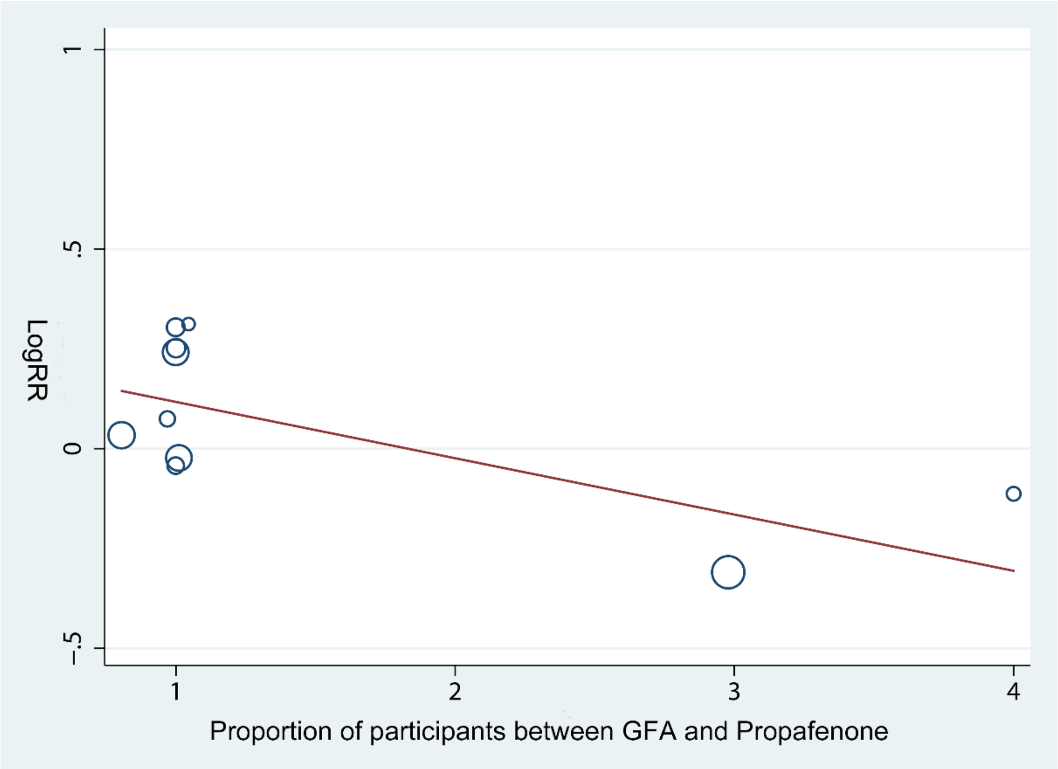

Supplementary Figure 2. Meta-regression bubble plots of the association between log RR and age. The size of each circle is inversely proportional to the variance of change.

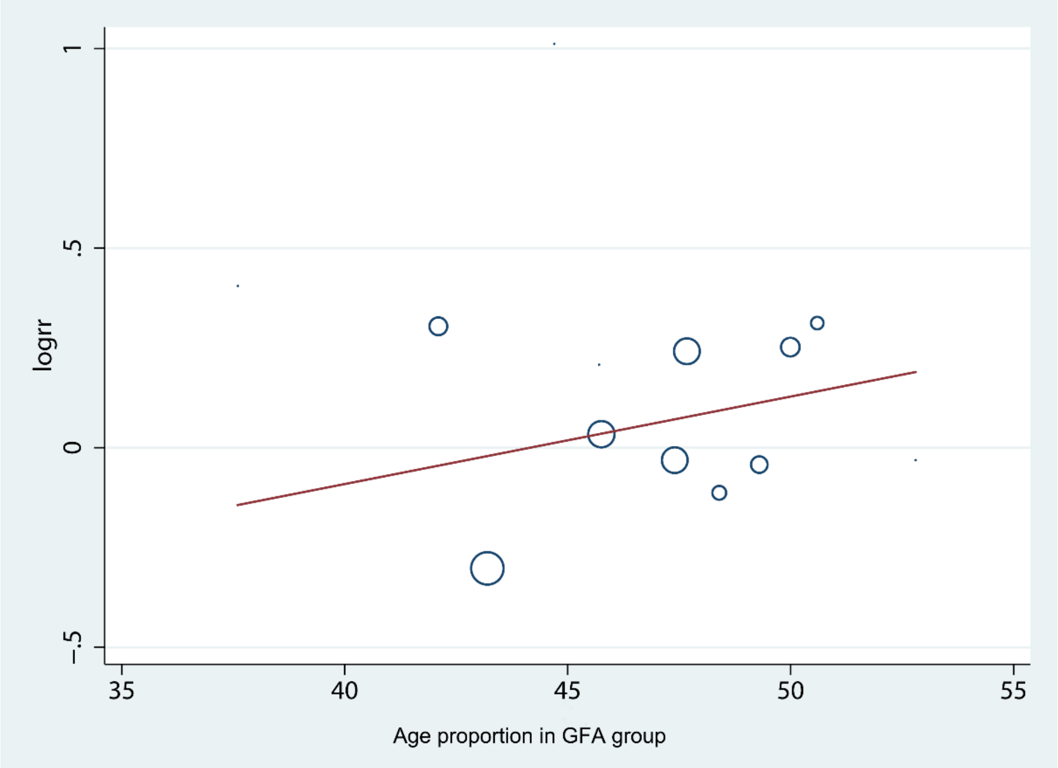

Supplementary Figure 3. Meta-regression bubble plots of the association between log RR and gender. The size of each circle is inversely proportional to the variance of change.

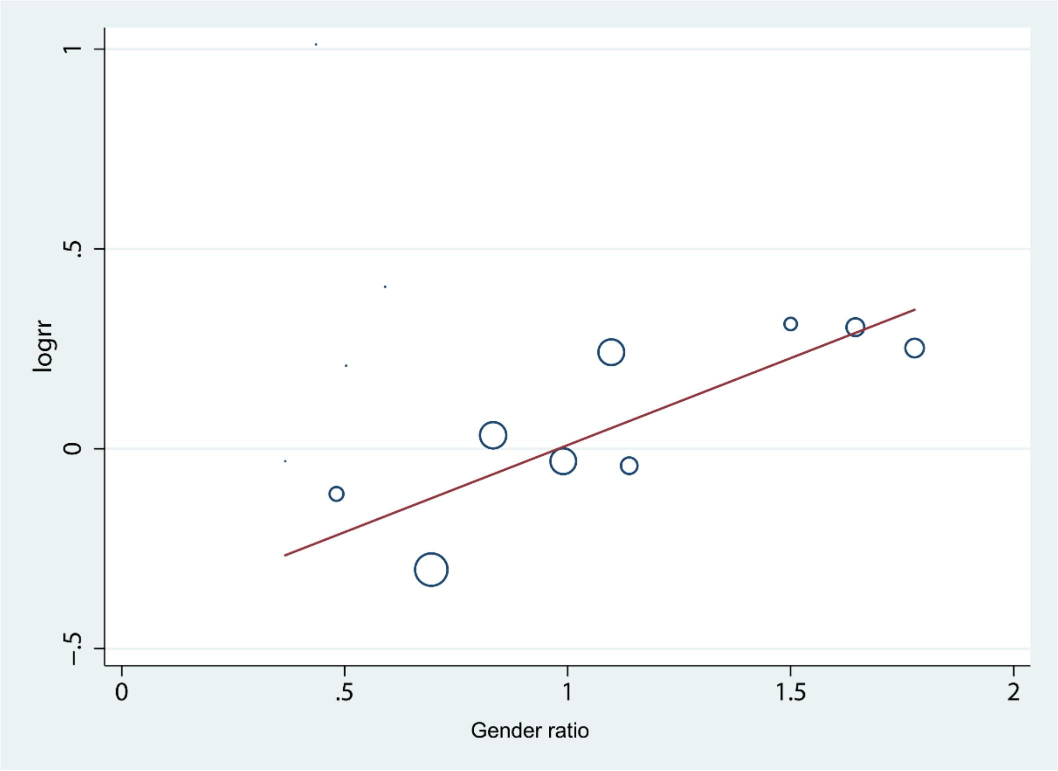

Supplementary Figure4. The leave-one-out method plot to assess heterogeneity in meta-analysis.

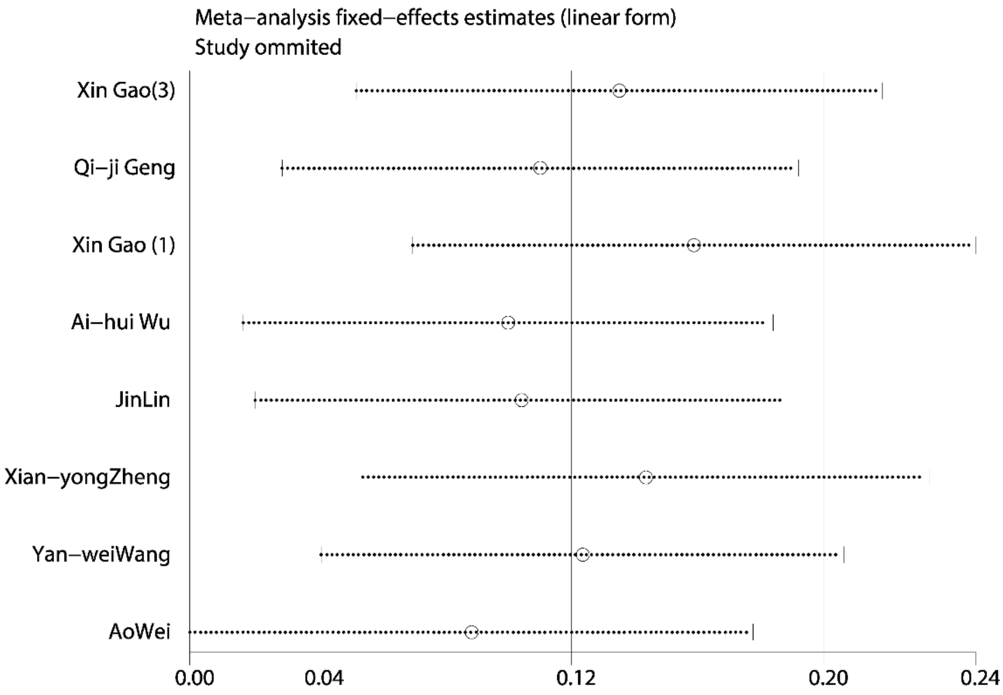

Supplementary Figure 5. Funnel plot detailing publication bias in the meta-analysis.

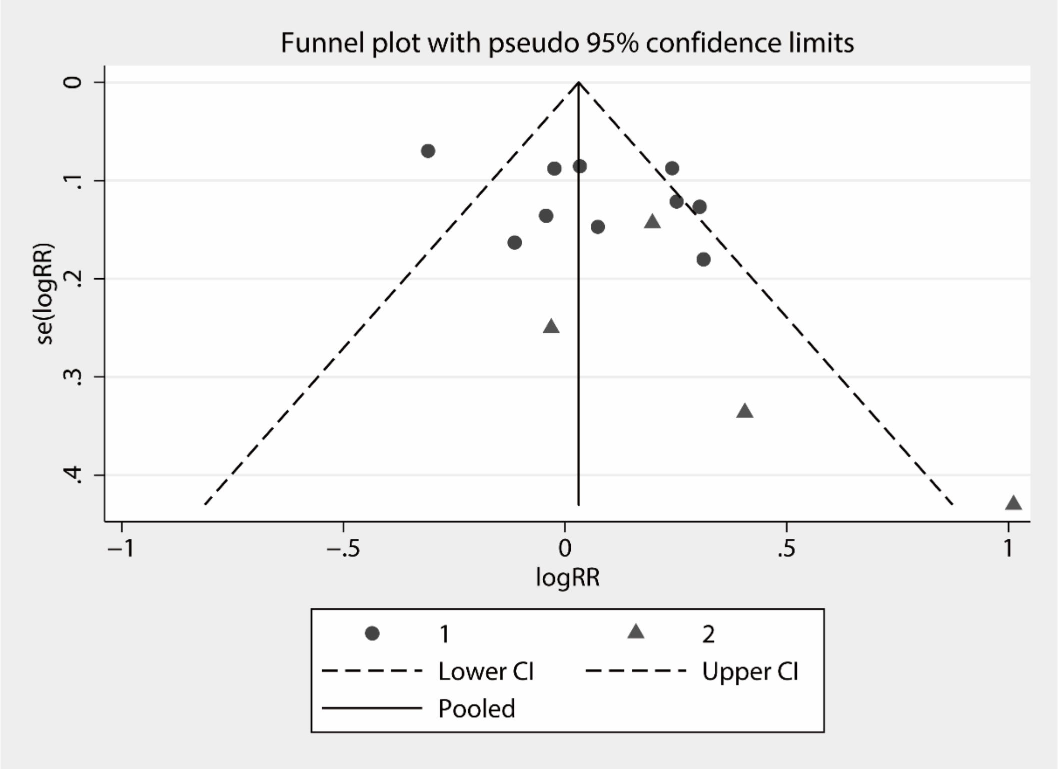

Supplementary Figure 6. Egger's publication bias plot for supraventricular tachycardia.

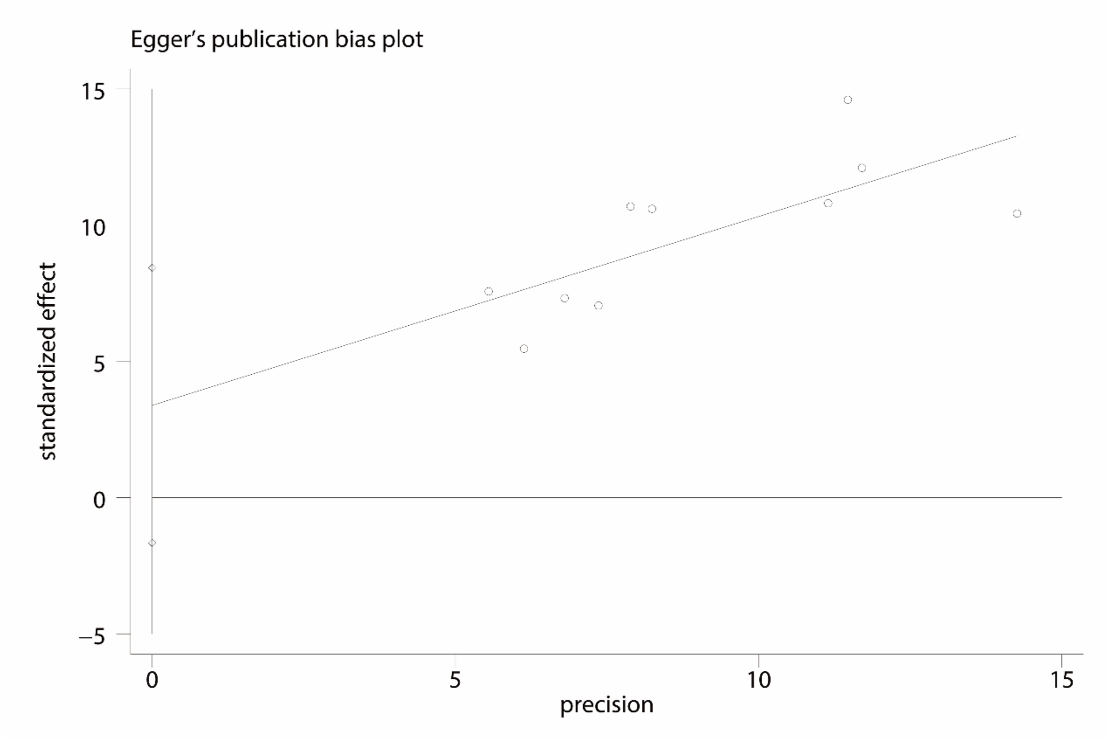

Supplementary Figure 7. Begg's test evaluate small study plot for supraventricular tachycardia.

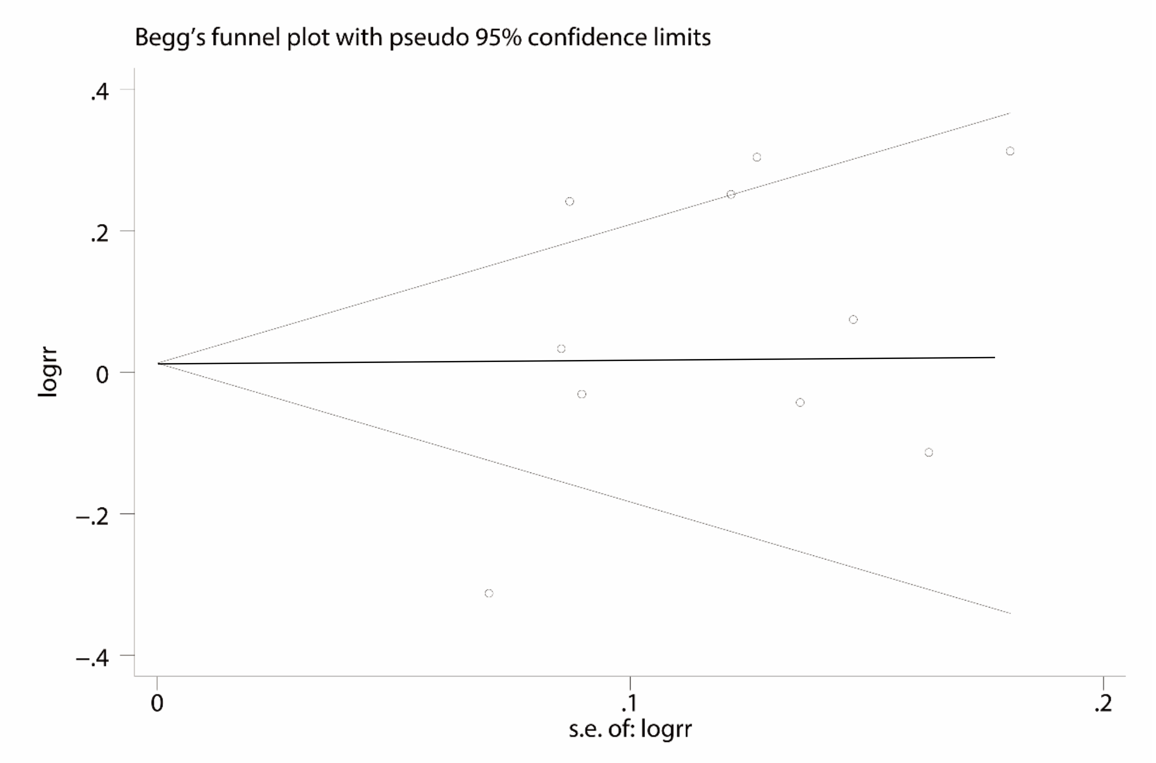

Supplementary Figure 8. Egger's publication bias plot for VPB.

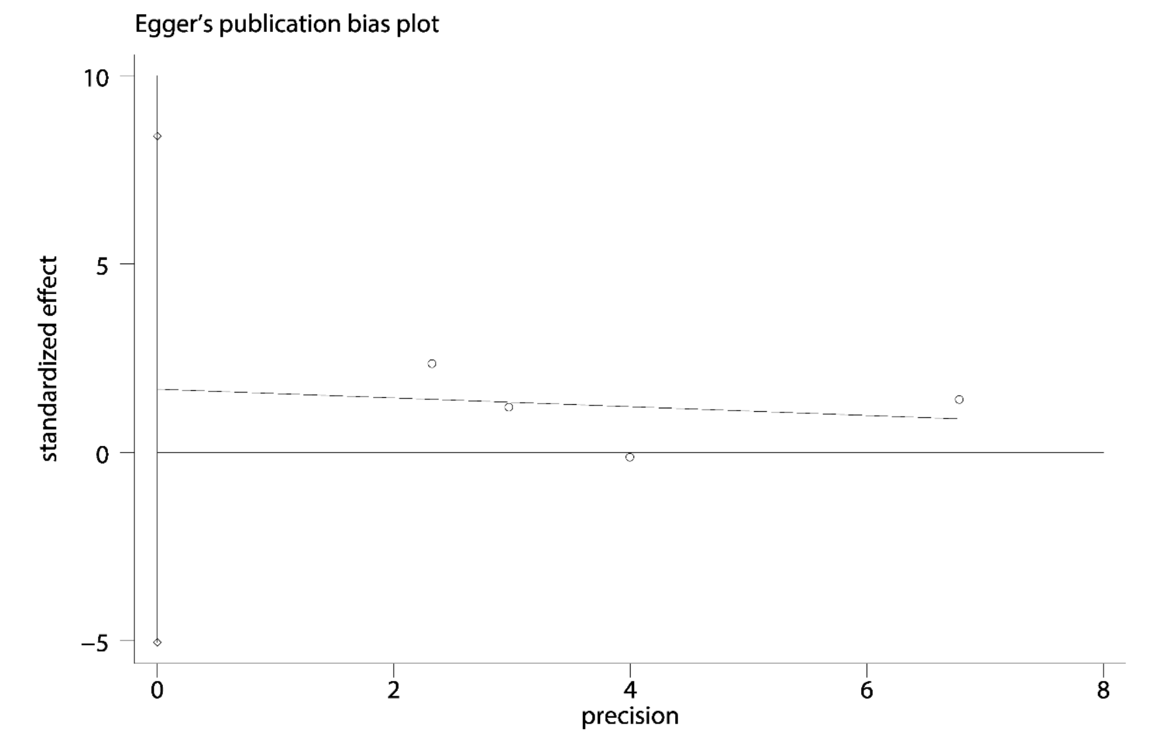

Supplementary Figure 9. Begg's test evaluate small study plot for VPB.

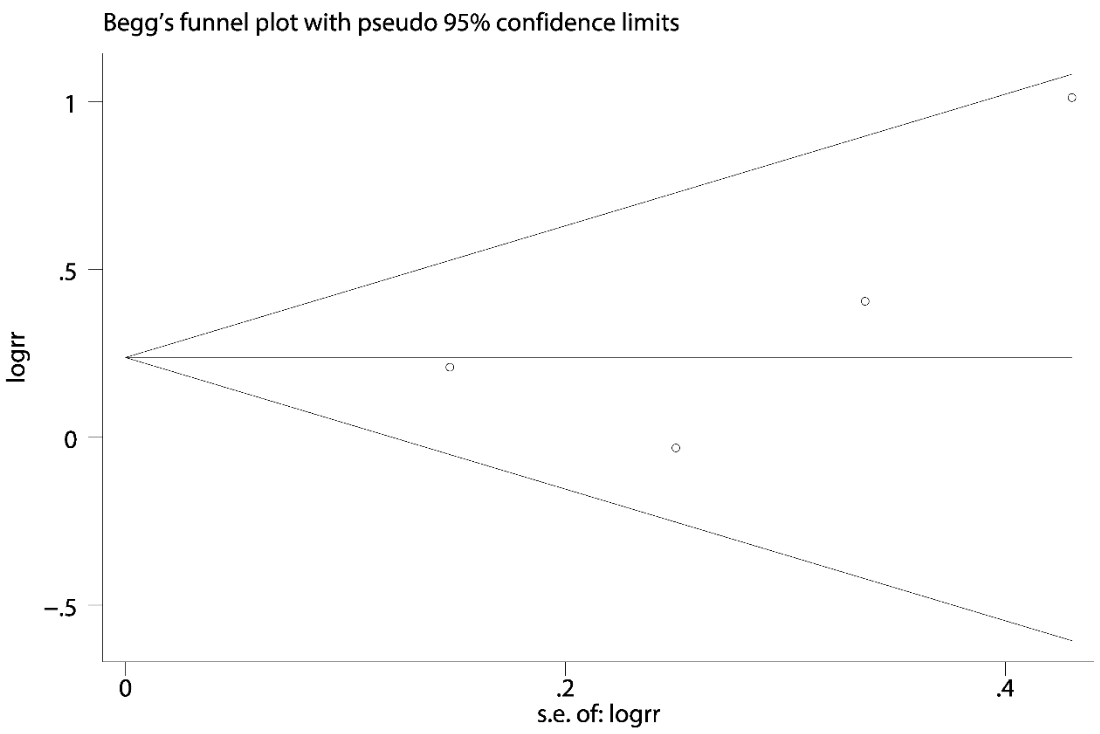

Supplementary Figure 10. "Trim and Fill" method to adjust publication bias for supraventricular arrhythmias.

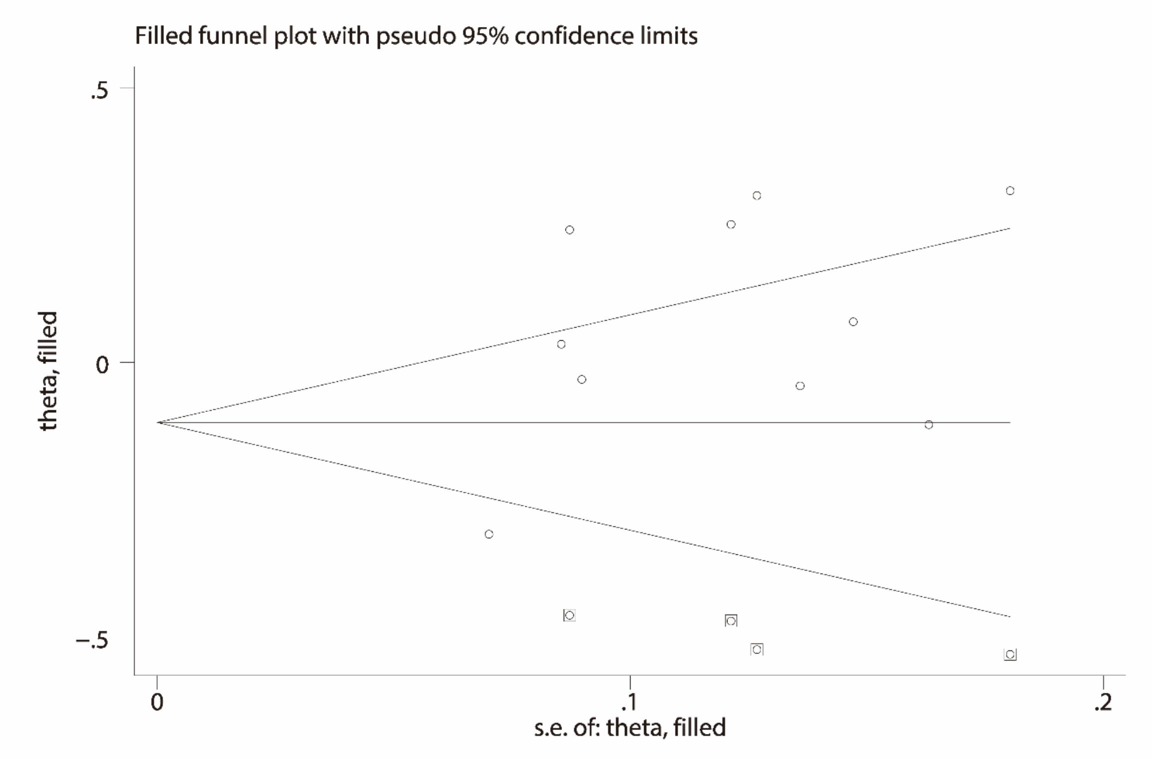

Supplementary Figure 11. “Trim and Fill” method to adjust publication bias for ventricular arrhythmias.

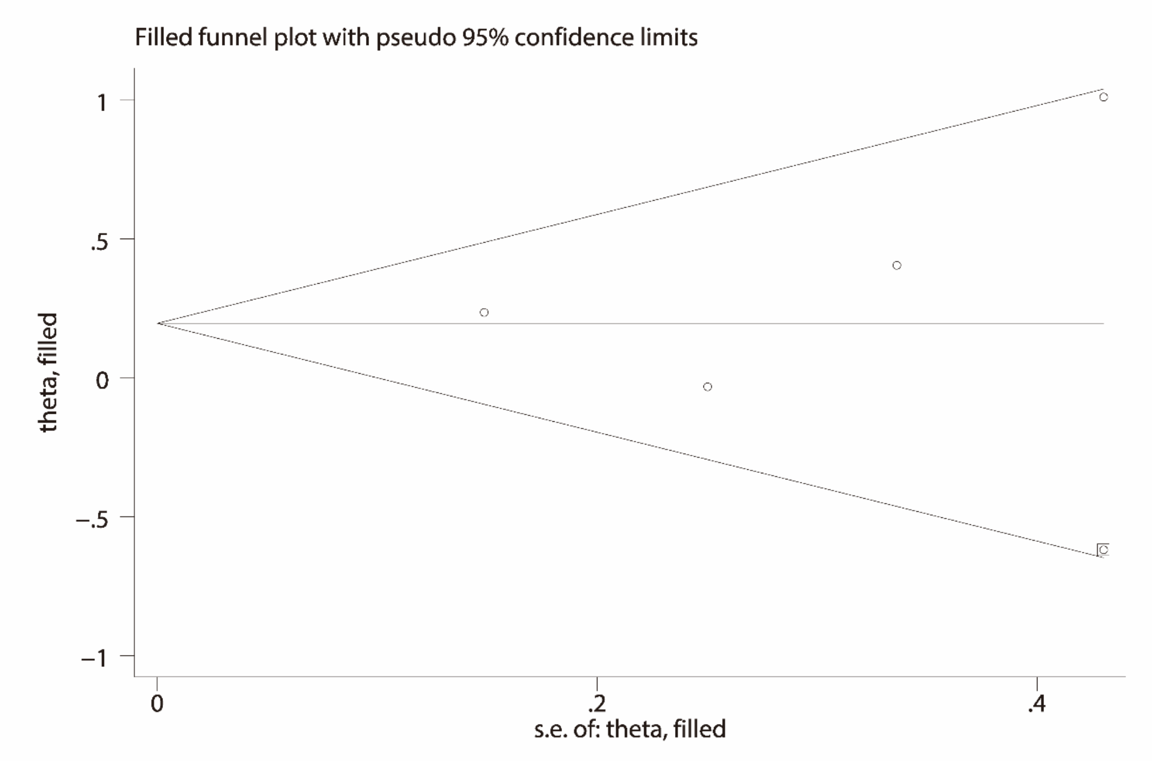

Supplementary Figure 12. – Funnel plot detailing publication bias in the meta-analysis with Systolic blood pressure.

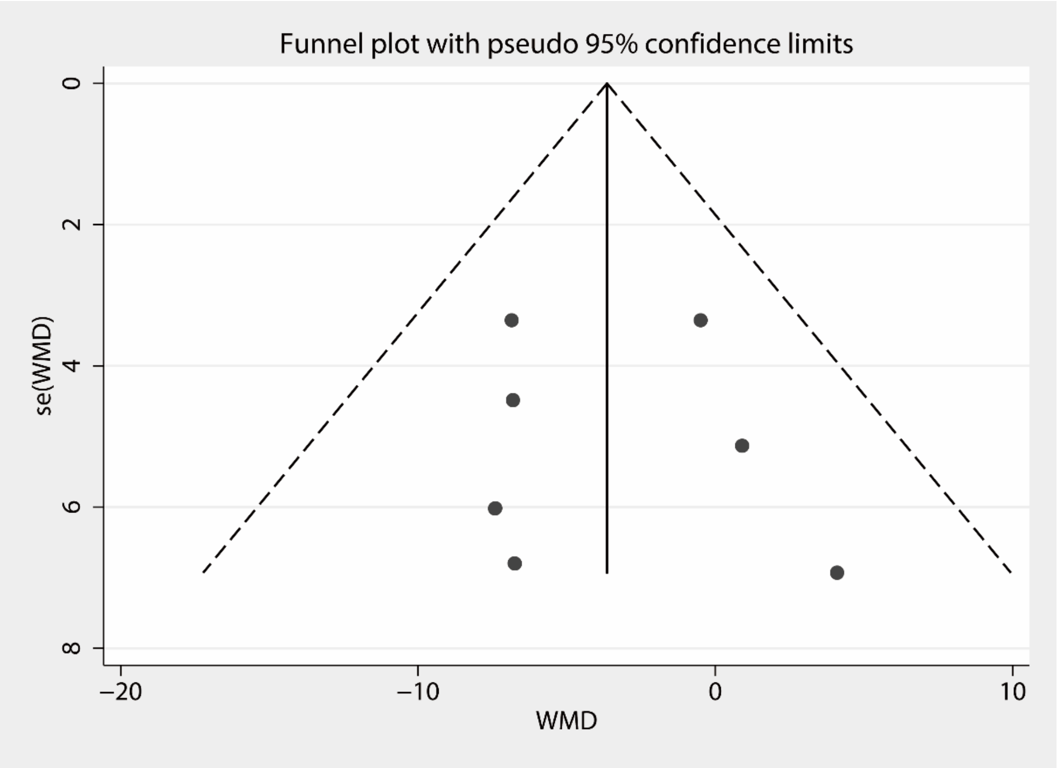

Supplementary Figure 13. – Funnel plot detailing publication bias in the meta-analysis with Diastolic blood pressure.

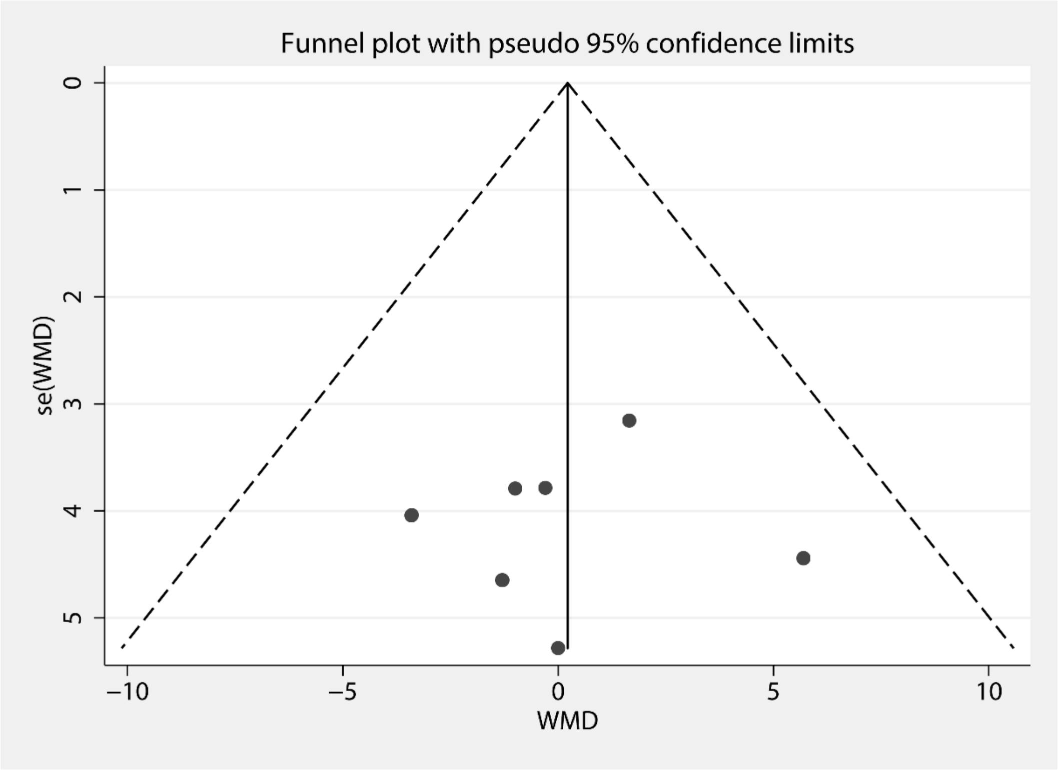

Supplementary Figure 14. Egger's publication bias plot with Systolic blood pressure.

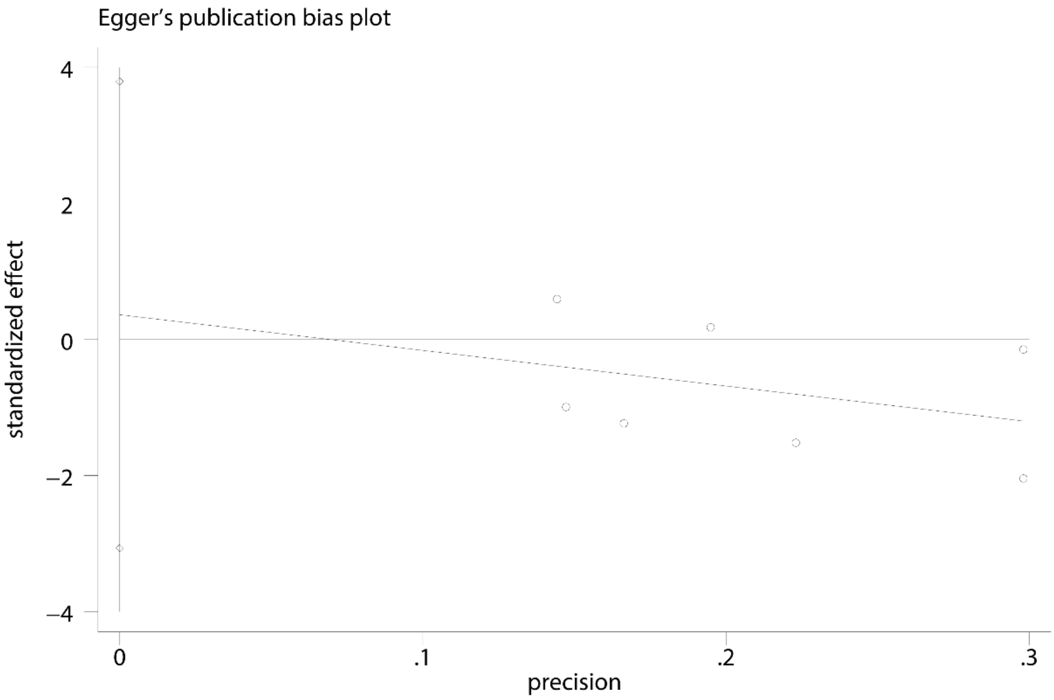

Supplementary Figure 15. Begg's publication bias plot with Systolic blood pressure.

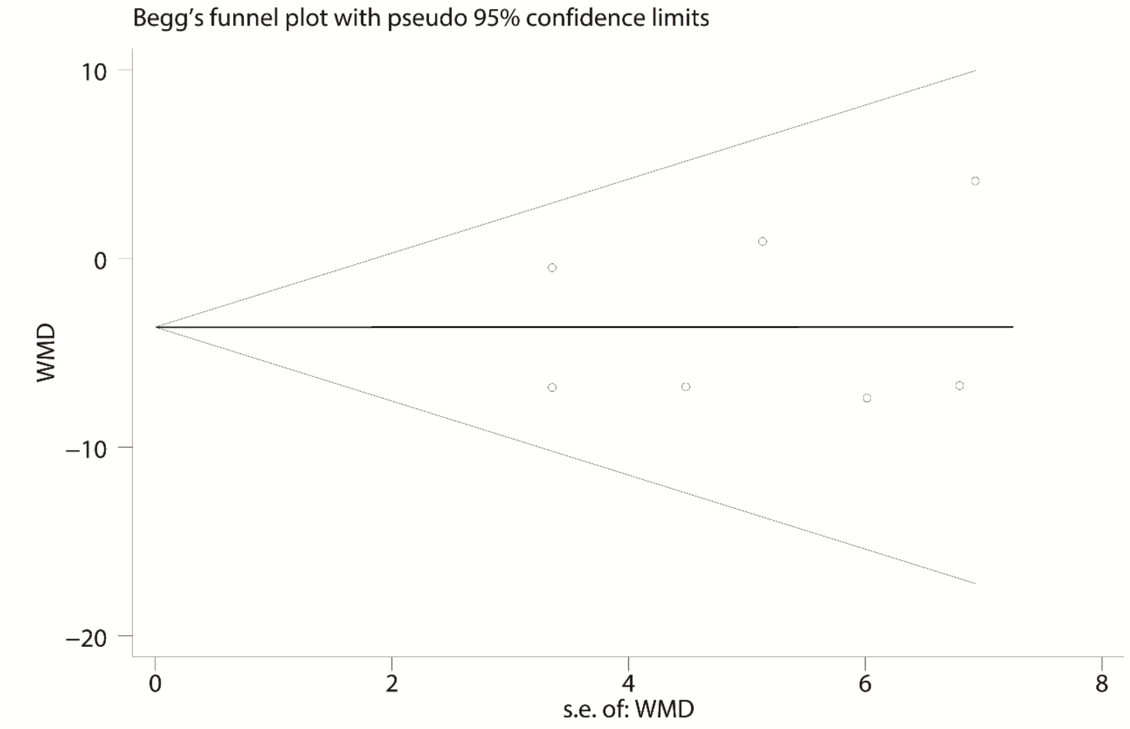

Supplementary Figure 16. Egger's publication bias plot with Diastolic blood pressure.

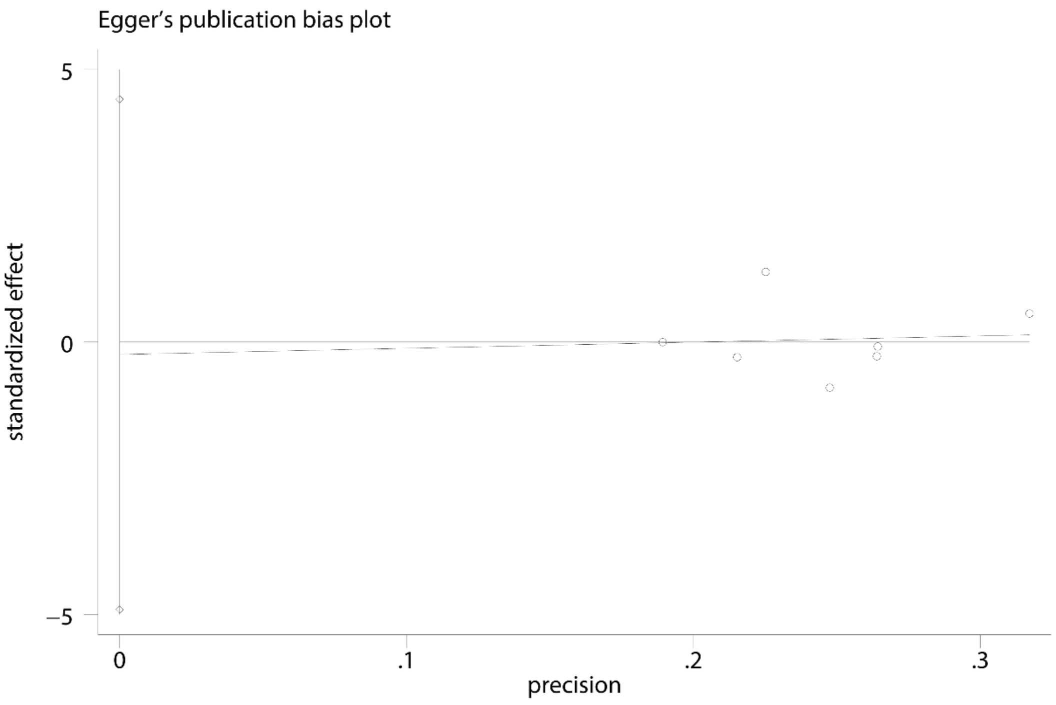

Supplementary Figure 17. Begg's publication bias plot with Diastolic blood pressure

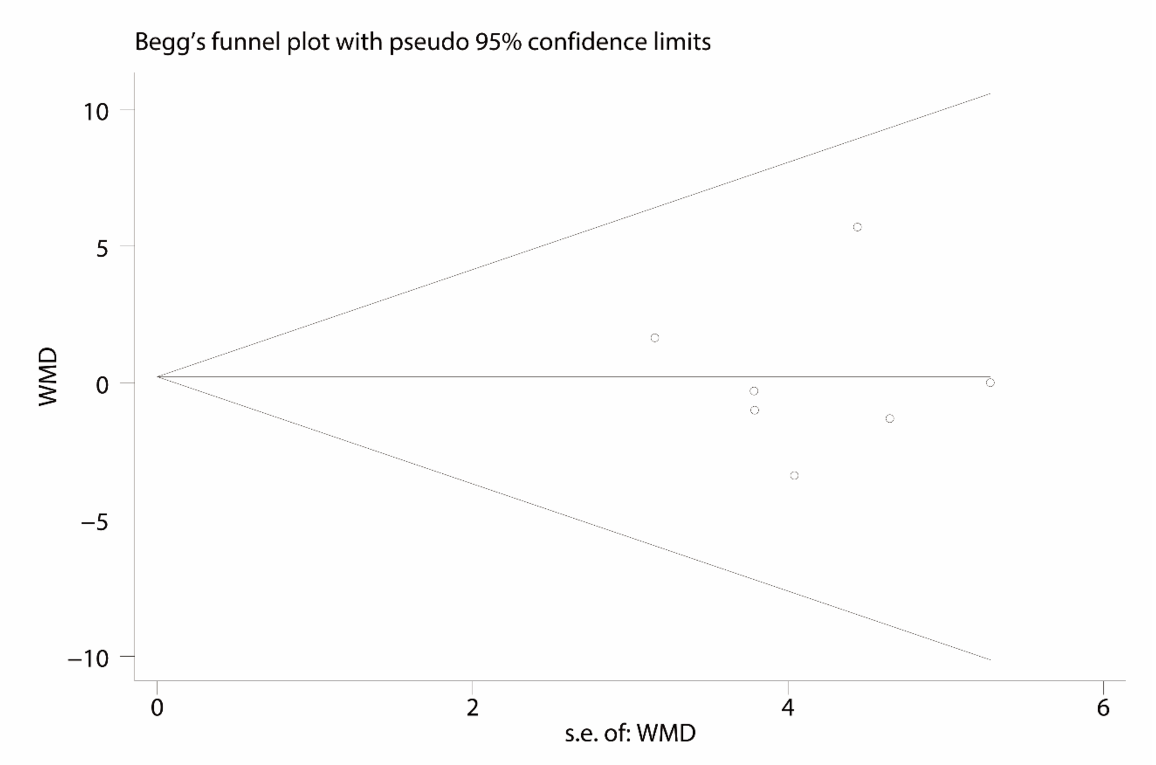

Supplementary Figure 18. Funnel plot detailing publication bias in the meta-analysis with HR.

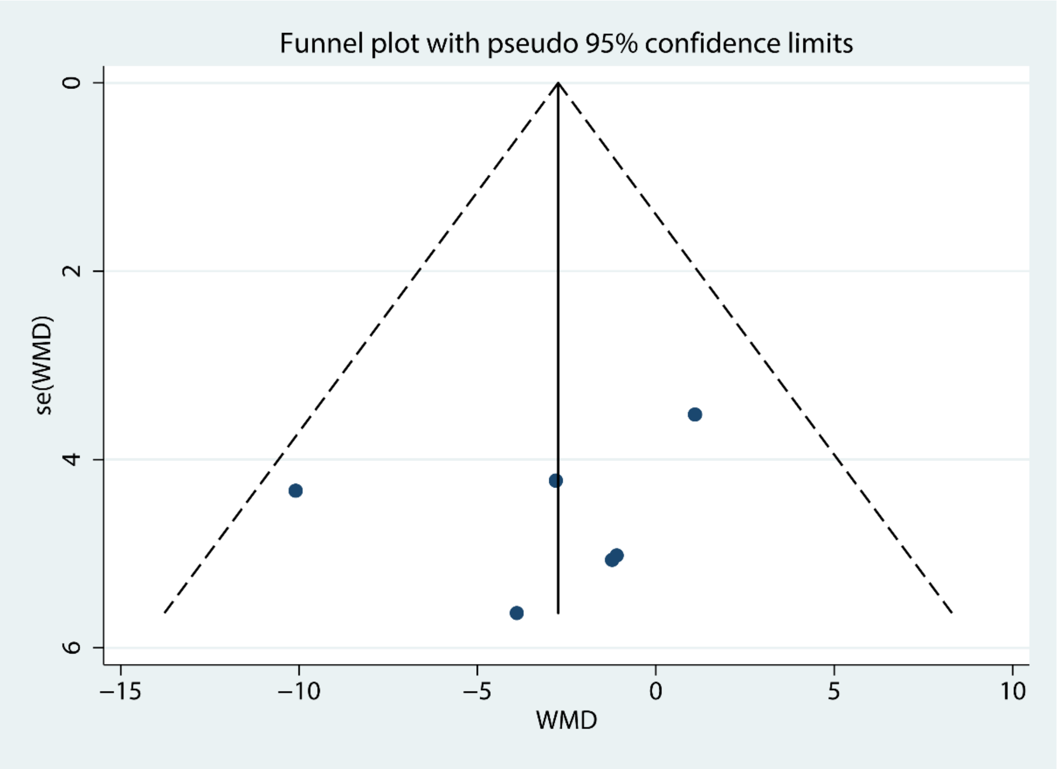

Supplementary Figure 19. Egger's publication bias plot with HR.

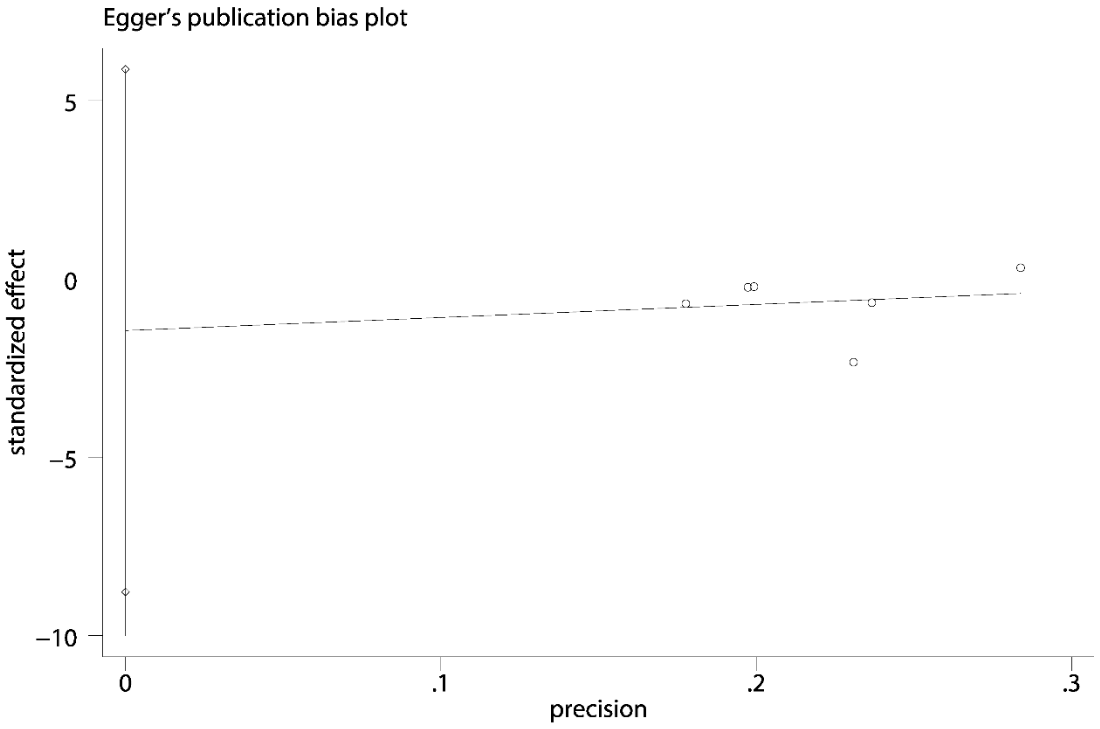

Supplementary Figure 20. Begg's publication bias plot with HR.

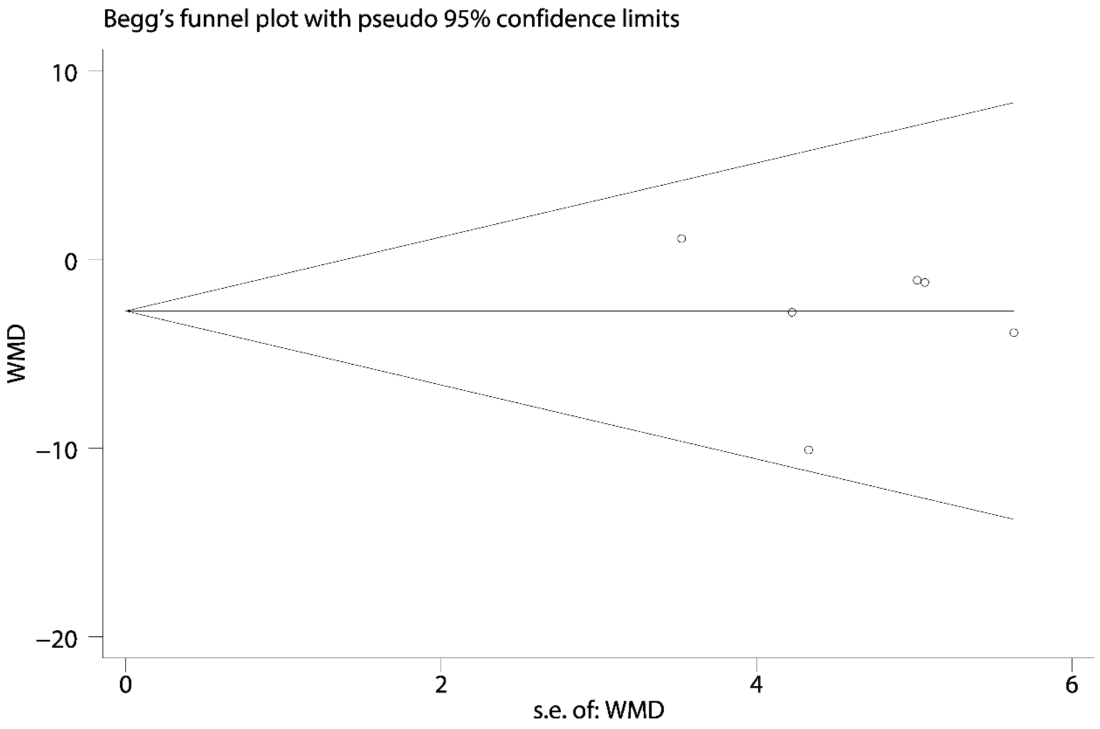

Supplementary Figure 21. Funnel plot detailing publication bias in the meta-analysis with PR.

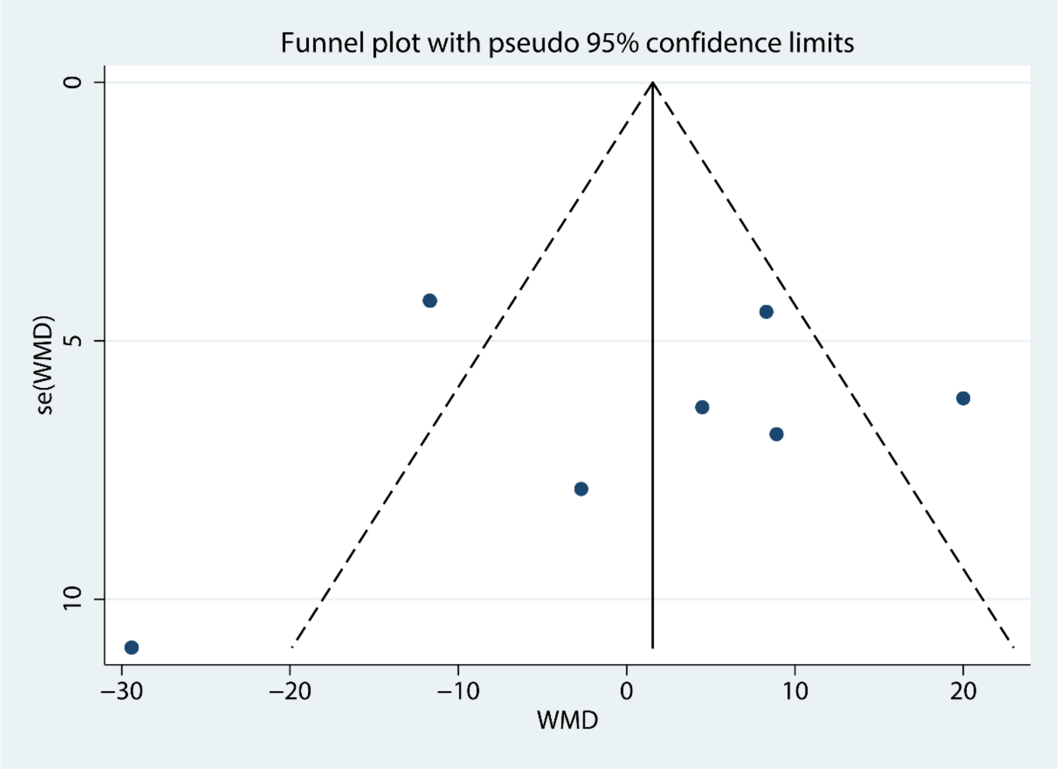

Supplementary Figure 22. Egger's publication bias plot with PR.

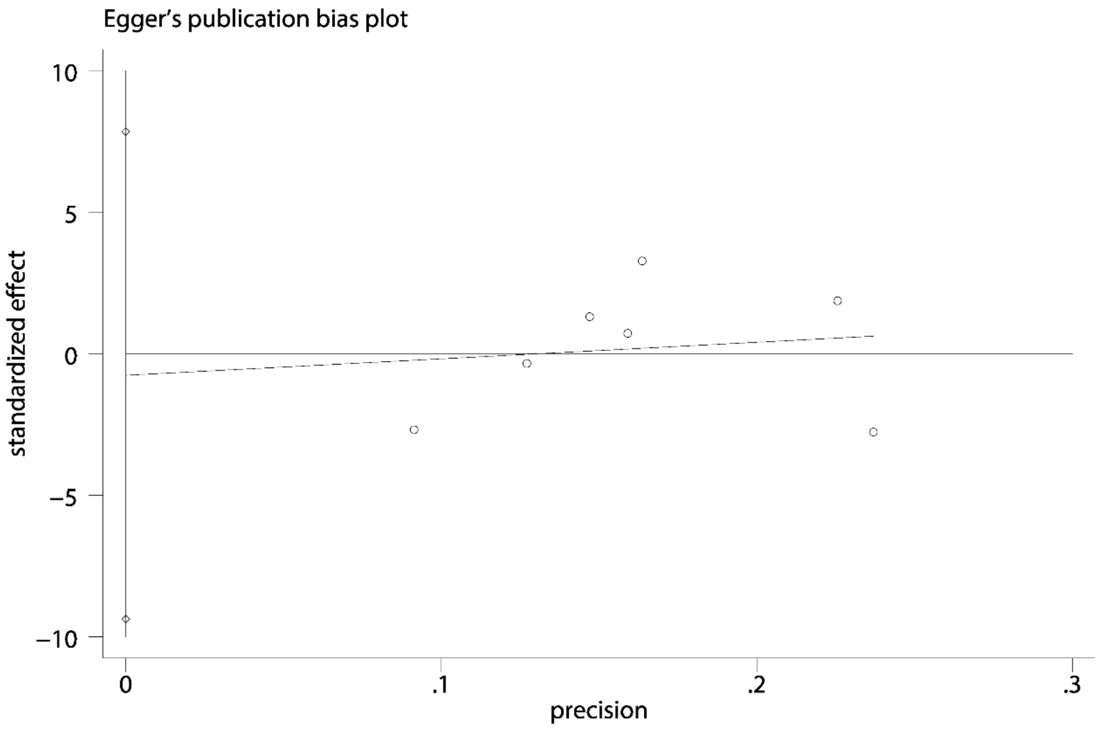

Supplementary Figure 23. Begg's publication bias plot with PR.

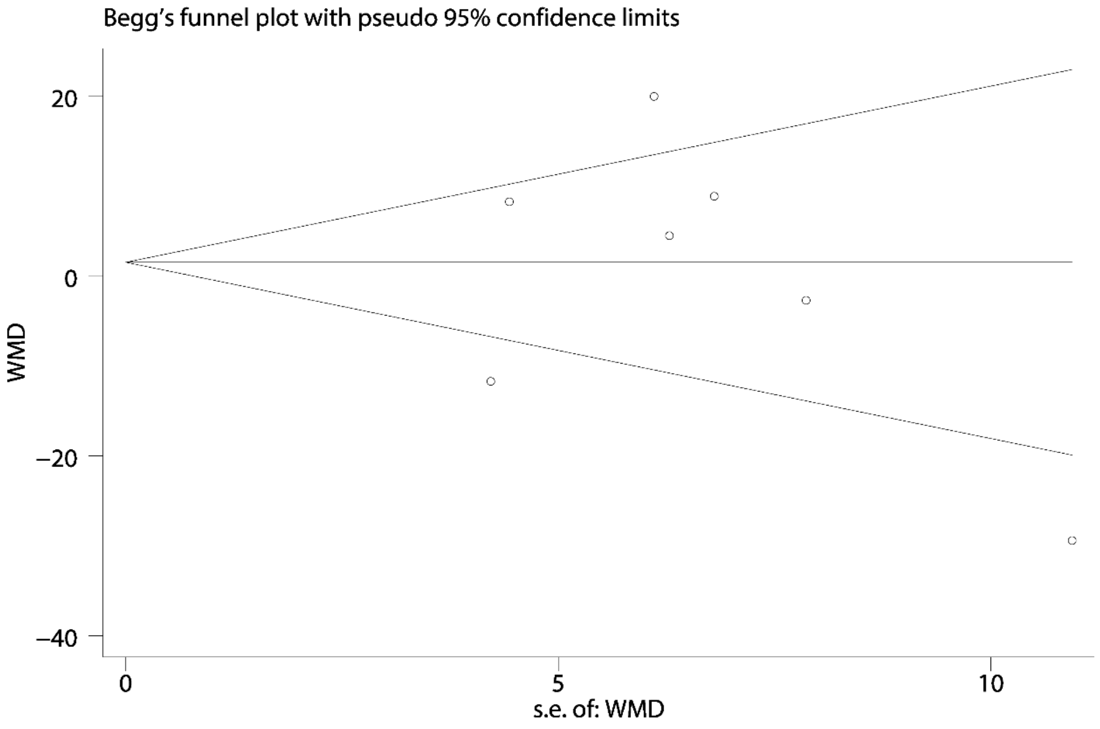

Supplementary Figure 24. Funnel plot detailing publication bias in the meta-analysis with QRS complex.

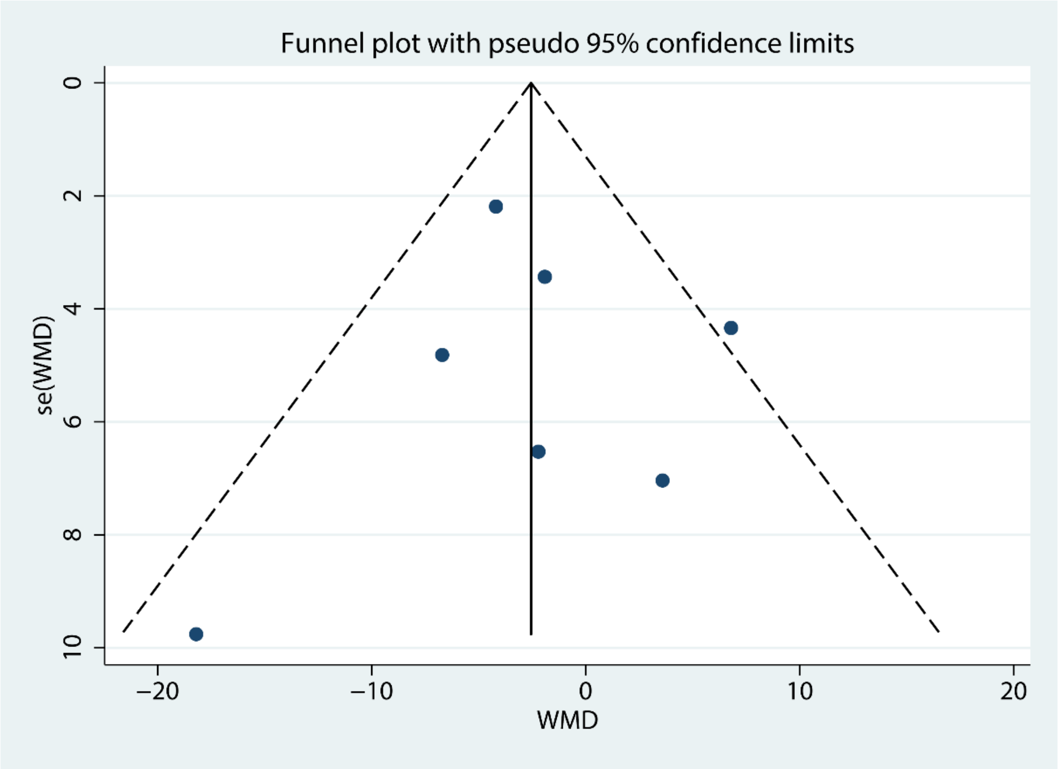

Supplementary Figure 25. Egger's publication bias plot with QRS complex.

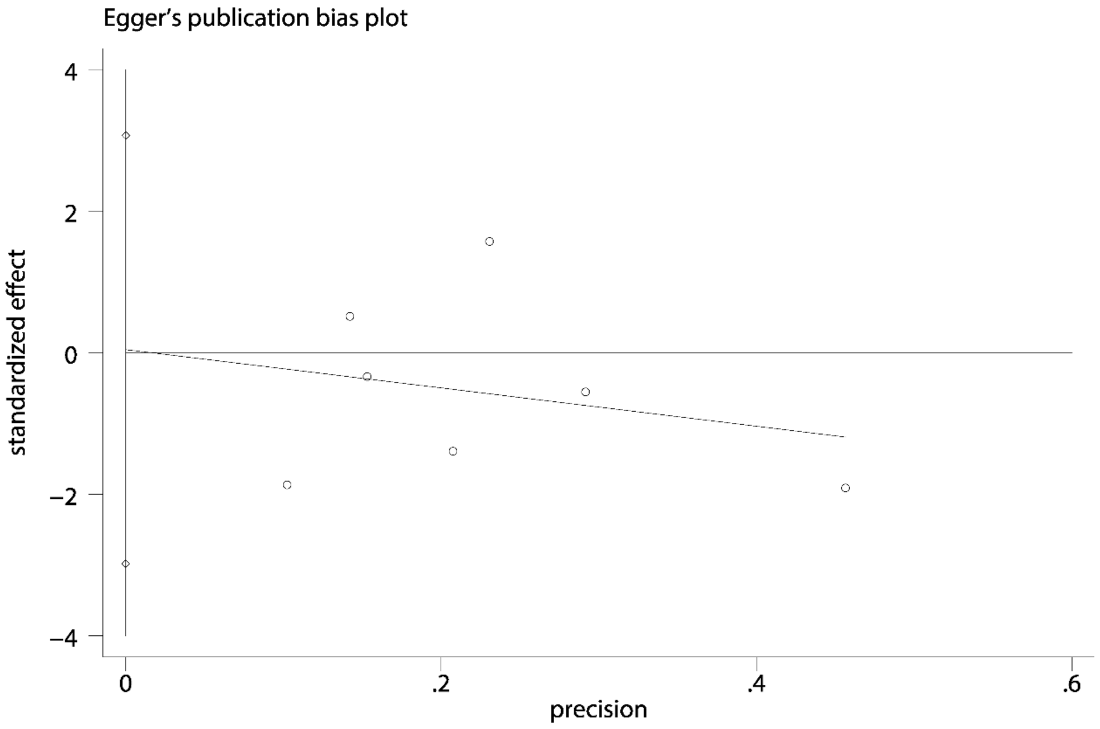

Supplementary Figure 26. Begg's publication bias plot with QRS complex.

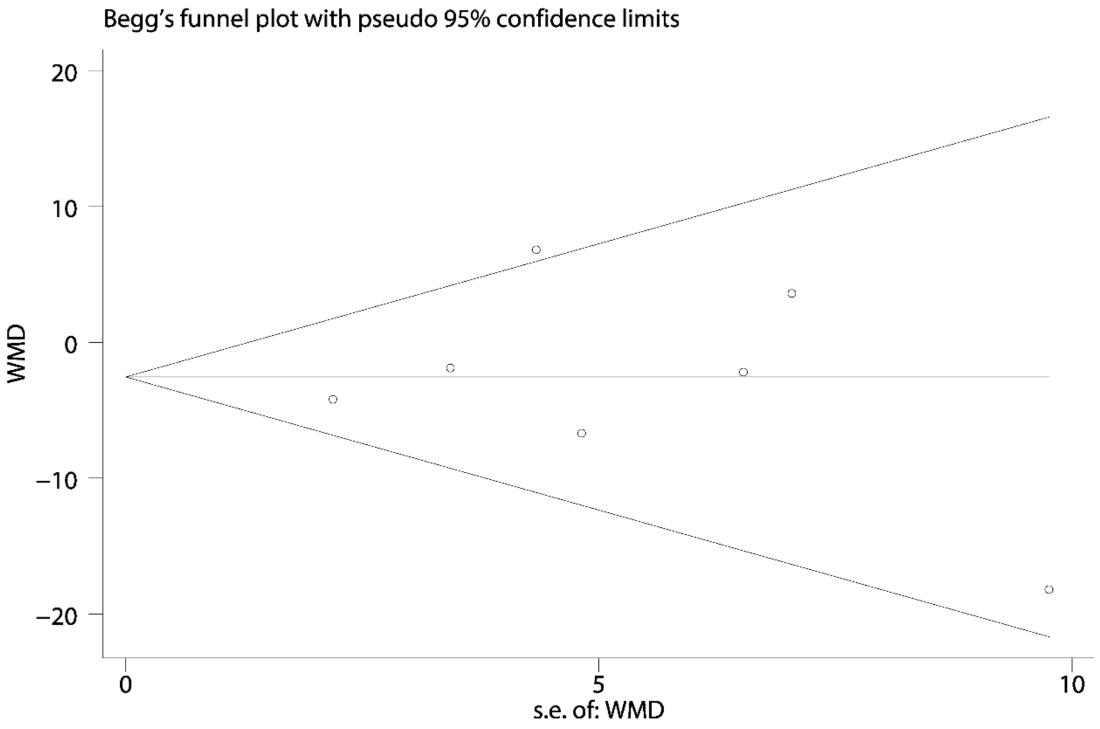

Supplementary Figure 27. Funnel plot detailing publication bias in the meta-analysis with QTc.

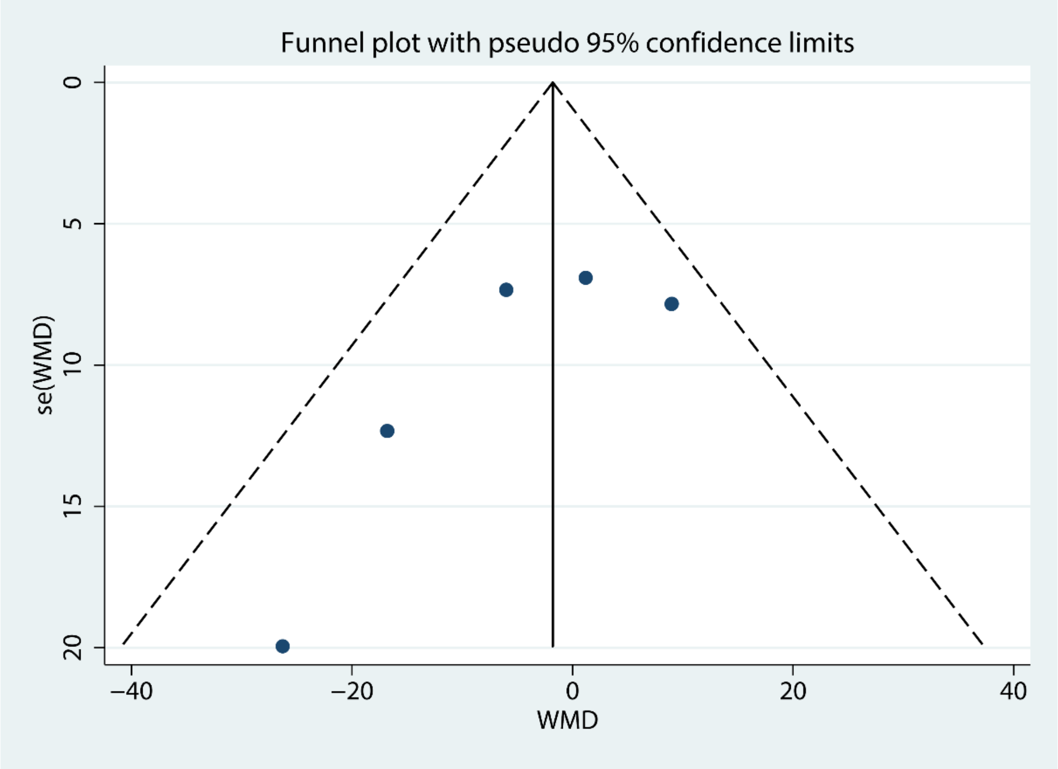

Supplementary Figure 28. Egger's publication bias plot with QTc.

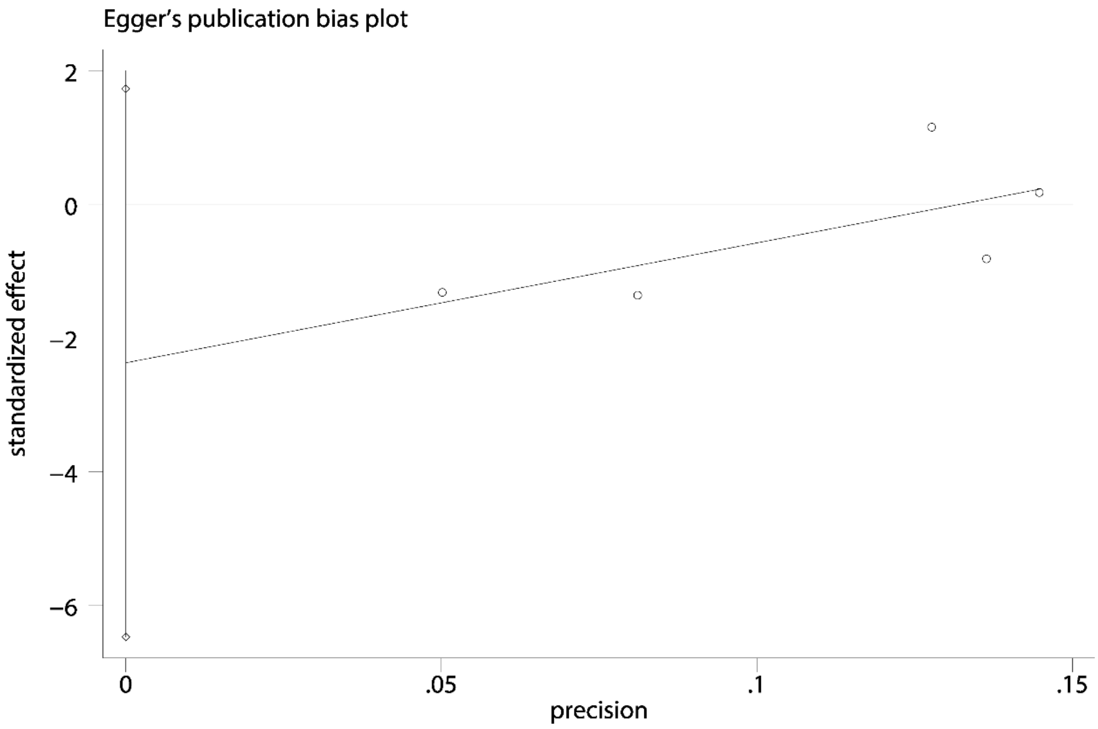

Supplementary Figure 29. Begg's publication bias plot with QTc.

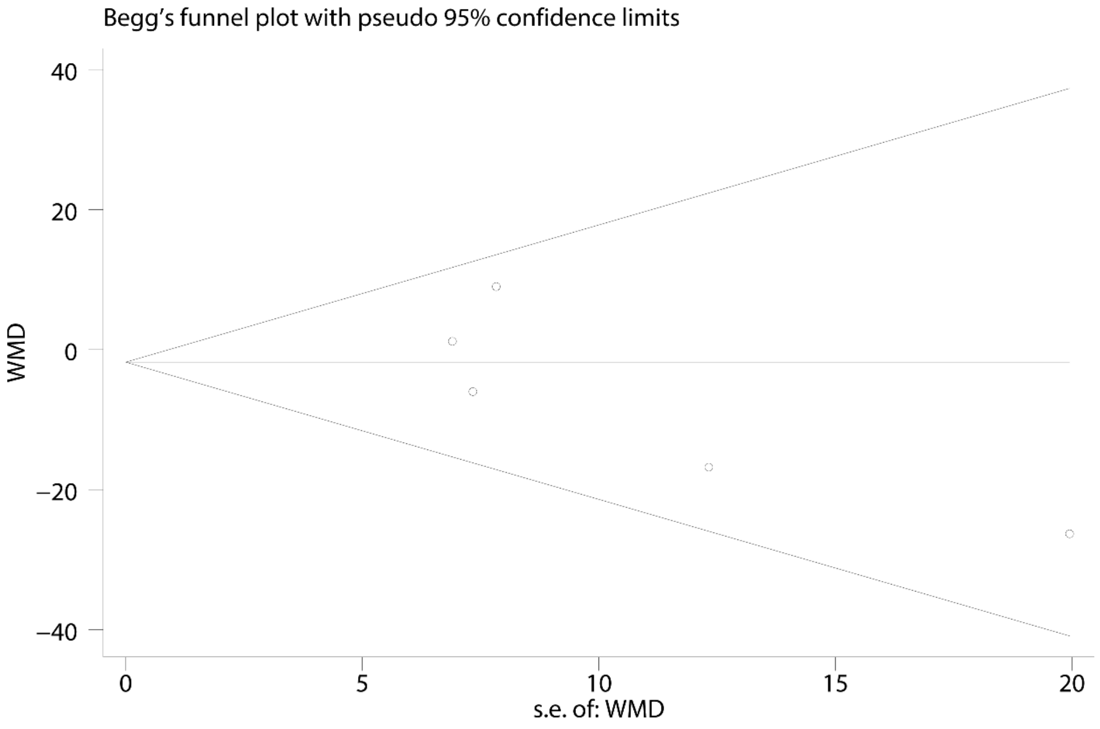

Supplementary Figure 30. Funnel plot detailing publication bias in the meta-analysis with mean converting time.

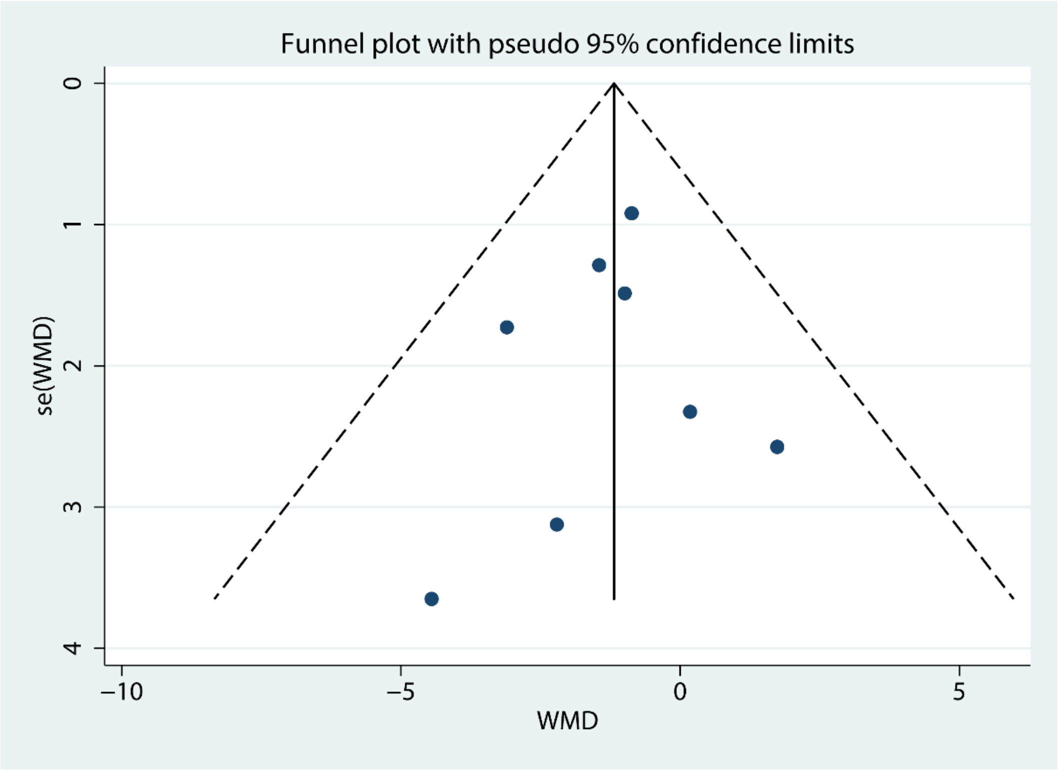

Supplementary Figure 31. Egger's publication bias plot with mean converting time.

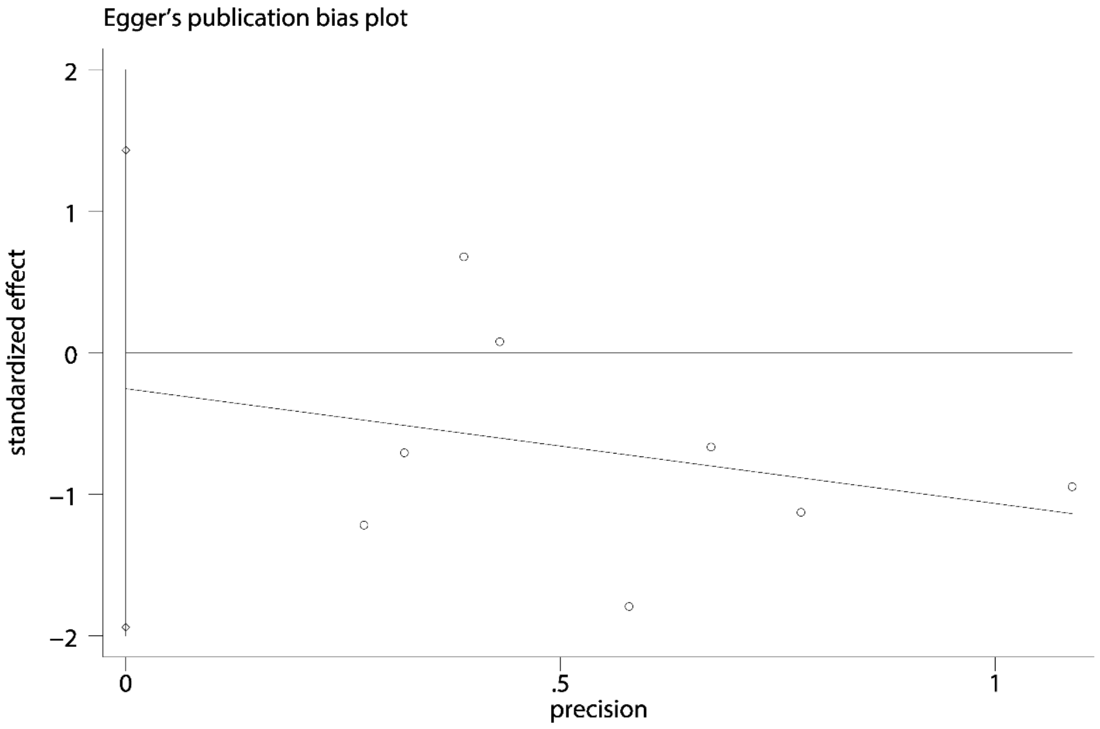

Supplementary Figure 32. Begg's publication bias plot with mean converting time.

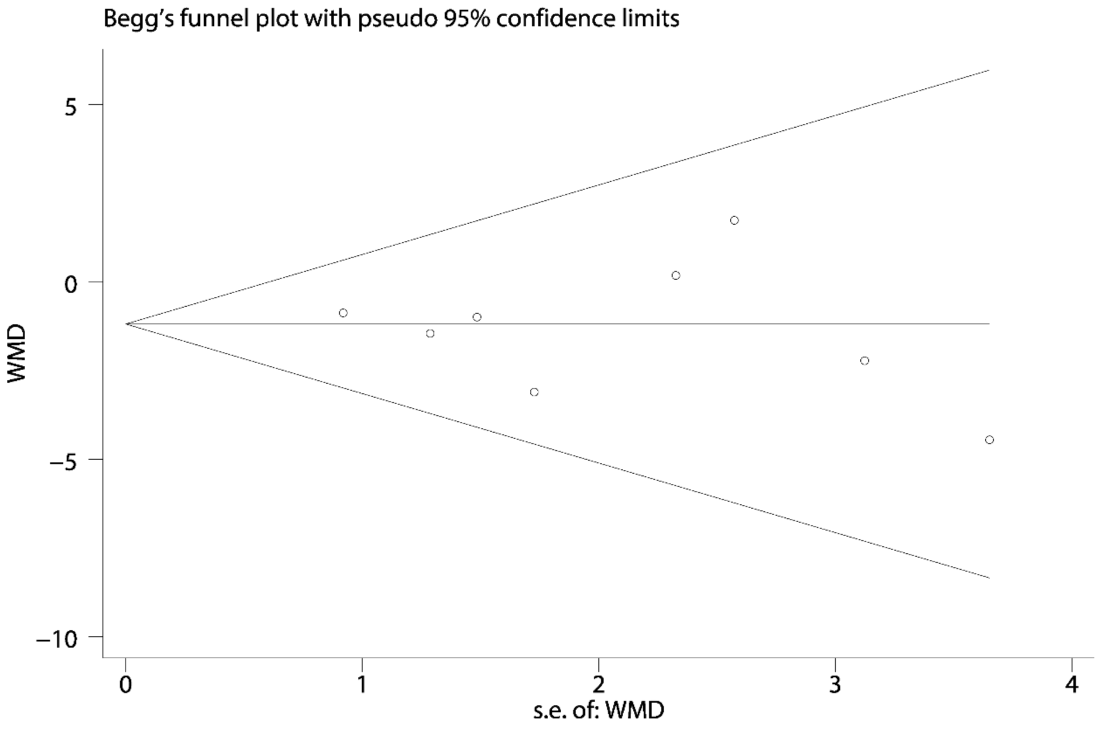

Supplementary Figure 33. Blood pressure compared before and after completed injection.

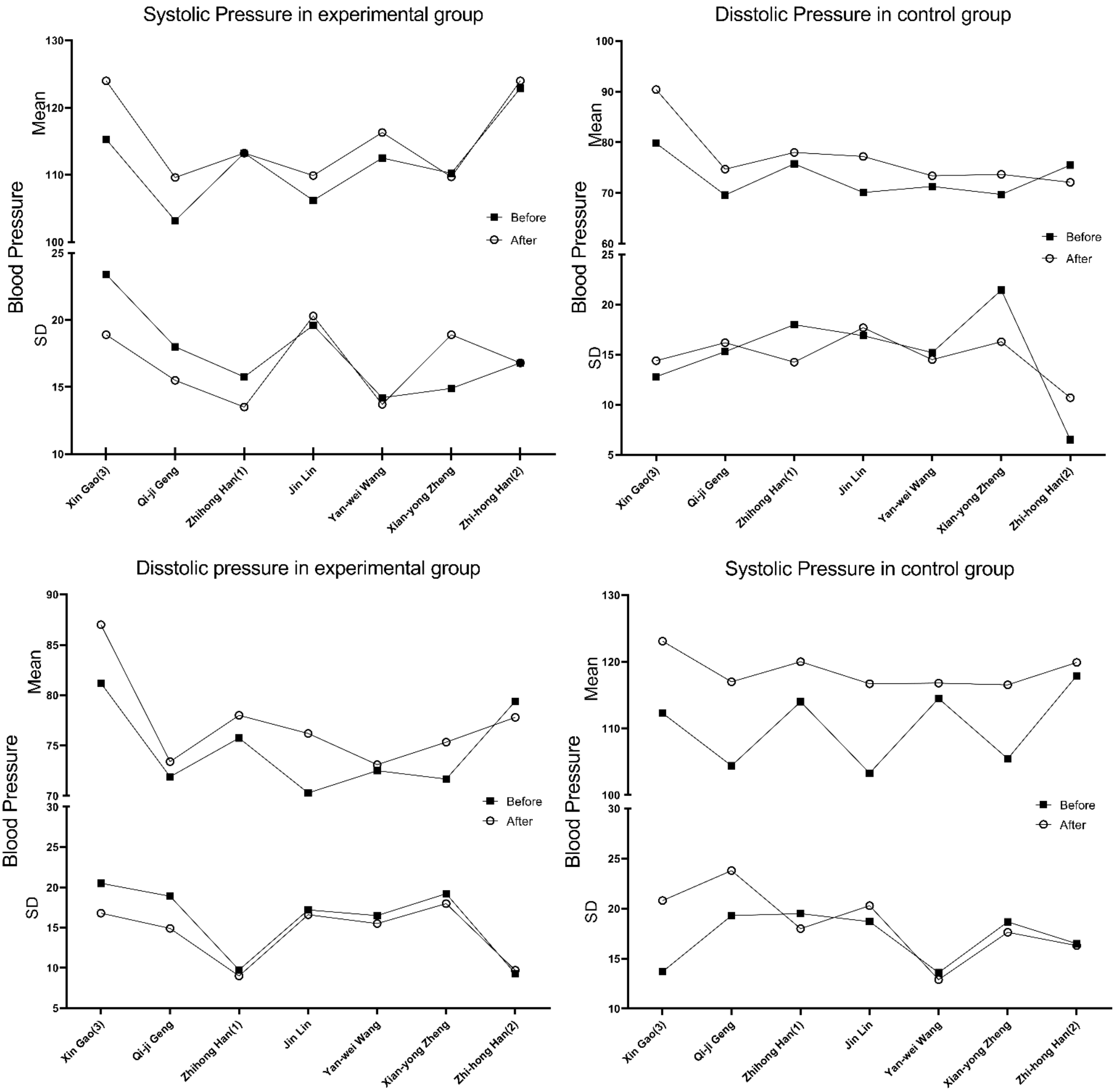

Other bias	Selective reporting	Incomplete outcome data	Blinding of participants, personal and outcome assessment	Allocation concealment	Random sequence generation	Year	author
No significant difference in baseline levels between the 2 groups	Unclear	no cases loss to follow-up	Identical volume and injection time	not motioned	Only refer to randomized trial in text, but without clear method	2003	Zhihong Han(1)
No significant difference in baseline (age, gender, stature, weight, course of disease, types of VPB, cardiothoracic ratio, left ventricular end diastolic diameter) levels between the 2 groups(p>0.05)	Unclear	2 patients withdrew before the beginning of the trial.1 is not eligible, the other disagreed with agreement.	Drugs injected in identical injector and duration of injection. Injection of drugs without the knowledge of either the patients or the researcher.	The third party(pharmacy) distributed drugs according to the random number	Professional staff generated random number by computer, patients get random number in order of enrollment.	2004	Xin Gao(3)
No significant difference in baseline(age) levels between the 2 groups	Unclear	no cases loss to follow-up	The drugs had the same shape specifications and was colorless and transparent injecting with selfsame 20ml injector.	The pharmacy are managed Drugs and distributed random number. Patients get random number in [illegible]	random number generate by computer, patients get random number in order of enrollment.	2006	Qiji Geng

Table 3. Details for assessment bias.

Other bias	Selective reporting	Incomplete outcome data	Blinding of participants personal and outcome	Allocation concealment	Random sequence generation	Year	author
No significant difference in baseline (gender) levels except age between the 2 groups. Research on innovative drugs and modernization of traditional Chinese medicine of the "863 Program" of the Ministry of Science and Technology of the People's Republic of China for a new class I drug against cardiac arrhythmias, GFA hydrochloride. (2002AA2Z3102)	Unclear	6 cases withdrew.1 because of severe side effect of GFA, others are unclear.	Drugs injected in identical injector and duration of injection.	Patients get random number in order of enrollment guiding by staff.	random number generate by professional staff who set up random number table. patients get random number in order of enrollment.	2007	Xin Gao (1)
No significant difference in baseline (age, gender, stature, weight, seizure frequency/year) levels between the 2 groups(p>0.05)."863 Program"(2002AA2Z3102)	Unclear	1 case withdraw (GFA). Failure to inject the second dose of medication as required.	Open-labeled	Patients get random number in order of selection.	Random number generate by professional staff who set up random number table, patients get random number in order of enrollment. The proportion of the number of people in the test group to the control group was 3:1.	2007	Xin Gao (2)
No significant difference in baseline levels between the 2 groups except age	Unclear	no cases loss to follow-up	Identical volume and injection time	Self-drawing a sealed envelope in the physician's sight.	Only refer to randomized trial in text, but without clear method	2010	Aihui Wu

Other bias	Selective reporting	Incomplete outcome data	Blinding of participants, personal and outcome assessment	Allocation concealment	Random sequence generation	Year	author
No significant difference in baseline levels between the 2 groups	Unclear	no cases loss to follow-up	Drugs injected in identical injector and duration of injection. Injection of drugs without the knowledge of either the patients or the researcher.	Third party not included in researchers and patients distributed drugs according to the random number.	random number generate by computer supervising by perfectional staff, patients get random number in order of enrollment.	2010	Jin Lin
No significant difference in baseline (age, gender, stature, weight, course of disease) levels between the 2 groups(p>0.05)	Unclear	no cases loss to follow-up	the injection solved in identical dextrose solution and identical injection method.	not motioned	without clear method	2012	Xianyong Zheng
No significant difference in baseline (age, gender, stature, weight, course of disease, types of SVP) levels between the 2 groups(p>0.05)	Unclear	no cases loss to follow-up	Identical volume and injection time	not motioned	random number generate by professional staff, patients get random number in order of enrollment.	2014	Yanwei Wang

Other bias	Selective reporting	Incomplete outcome data	Blinding of participants and personal outcome assessment	Allocation concealment	Random sequence generation	Year	author
Not motioned	Unclear	no cases loss to follow-up	Identical volume and injection time	not motioned	divided 86 cases into two groups according to random number generator.	2019	Ao Wei
No significant difference in baseline (age, gender, course of disease) levels between the 2 groups(p>0.05)	Unclear	no cases loss to follow-up	Drugs have same dosage form.	not motioned	Only motioned randomize number, but without clear method.	2003	Zhihong Han (2)
No significant difference in baseline levels between the 2 groups	Unclear	no cases loss to follow-up	Drugs solved in identical solution and injected in identical injector.	Self-drawing a sealed envelope in the physician's sight.	The patient draws a sealed random envelope based on generating a random number	2004	Lianfang Chang

Other bias	Selective reporting	Incomplete outcome data	Blinding of participants and personal.<br>outcome assessment	Allocation concealment	Random sequence generation	Year	author
No significant difference in baseline (age, gender, course of disease) levels between the 2 groups($p>0.05$)	Unclear	no cases loss to follow-up	Drugs injected in identical injector and duration of injection.	The third party(pharmacy) distributed drugs according to the random number	Professional staff generated random number by computer, patients get random number in order of enrollment.	2004	Xin Gao (4)
No significant difference in baseline (age, gender, Premature ventricular contractions/24h) levels between the 2 groups($p>0.05$)	Unclear	3 cases withdraw. 2 dizziness; hand and tongue numbness (GFA). 1 nausea and dizziness (Propafenone).	Open-labeled	Staff on the basis of sealed envelope distributed drugs.	Randomization was based on simple computer-generated random digits.	2006	Yanmin Yang

Table 3. Details for assessment bias.
